# Electrochemically Promoted Benzylation of [60]Fullerooxazolidinone

**DOI:** 10.3390/nano12132281

**Published:** 2022-07-01

**Authors:** Xing-Xing Yan, Chuang Niu, Shi-Qi Ye, Bo-Chen Zhao, Guan-Wu Wang

**Affiliations:** 1 Hefei National Research Center for Physical Sciences at the Microscale and Department of Chemistry, University of Science and Technology of China, Hefei 230026, China; yxx1122@mail.ustc.edu.cn (X.-X.Y.); cniu@mail.ustc.edu.cn (C.N.); yeshiqi@mail.ustc.edu.cn (S.-Q.Y.); zbc1946974933@mail.ustc.edu.cn (B.-C.Z.); 2 State Key Laboratory of Applied Organic Chemistry, Lanzhou University, Lanzhou 730000, China

**Keywords:** [60]fullerene, electrochemistry, [60]fullerooxazolidinone, benzylation, addition patterns

## Abstract

Benzylation of the electrochemically generated dianion from *N*-*p*-tolyl-[60]fullerooxazolidinone with benzyl bromide provides three products with different addition patterns. The product distribution can be dramatically altered by varying the reaction conditions. Based on spectral characterizations, these products have been assigned as mono-benzylated 1,4-adduct and bis-benzylated 1,2,3,16- and 1,4,9,25-adducts, respectively. The assigned 1,2,3,16-adduct has been further established by X-ray diffraction analysis. It is believed that the 1,4-adduct is obtained by decarboxylative benzylation of the dianionic species, while bis-benzylated 1,2,3,16- and 1,4,9,25-adducts are achieved via a rearrangement process. In addition, the electrochemical properties of these products have been studied.

## 1. Introduction

Functionalization of [60]fullerenes (C_60_) has attracted increasing attention due to their potential applications in biomedical sciences [1,2,3], materials [4,5,6], and perovskite solar cells (PSCs) [7,8,9,10,11]. Scientists have established diverse approaches for the synthesis of various fullerene derivatives [12,13,14,15,16,17,18]. Among them, electrosynthesis has been demonstrated as an efficient and selective method for synthesizing functionalized fullerene derivatives [19,20,21,22,23,24,25].

Recently, the C_60_-fused heterocyclic derivatives have been frequently used in electrosynthesis, because upon acceptance of electrons, the cleavage of carbon-heteroatom bond in the substrates induces the rearrangement of the fused heterocycle from a [6,6]-bond to a [5,6]-bond and affords novel fullerene derivatives with new addition patterns. Fulleroindolines, as representative fullerene derivatives, have been systematically applied in electrochemical synthesis. Our group demonstrated the electrosynthesis of the 1,2,3,16-adducts from fulleroindoline and alkyl bromides, acyl chlorides, or chloroformates [26,27]. Subsequently, we reported the electrochemical reactions of the fulleroindolines with 1,2-bis(bromomethyl)benzene or phthaloyl chloride, which regioselectively provided 1,2,4,17-adducts [28,29], while the bulky alkyl bromides, such as 2,4,6-tris(bromomethyl)mesitylene and diphenylbromomethane, were also exploited to functionalize fulleroindolines, and afforded 1,2,3,16-adducts and a rare 1,4,9,12-adduct [30]. All of these reactions involved the rearrangement of the indoline moiety from a [6,6]-bond to a [5,6]-bond. Similar electrosynthesis involving rearrangements of heterocyclic moieties on the fullerene surface for other C_60_-fused heterocycles resulted in 1,2,3,4-, 1,2,3,16- and 1,4,9,25-adducts [31,32,33,34,35]. The electrochemical reactions of heterocyclic fullerene derivatives fused with two heteroatoms have also been reported. The Gao group reported the reaction of dianionic fullerooxazolines with benzyl bromide (BnBr) and successfully obtained a 1,2,3,4-adduct [36]. Later, they obtained two 1,2,3,16-adduct isomers and one 1,2,3,4-adduct isomer by reductive benzylation of fulleroimidazoline [37]. In 2015, Gao and coworkers obtained 1,2,3,16-adducts by electrochemical benzylation of singly bonded 1,2,4,15-C_60_ dimers with an oxazoline or imidazoline moiety [38]. Among the various fullerene derivatives, fullerooxazolidinone derivatives are of interest because the carbamic moiety is fused to the fullerene skeleton via two heteroatoms. We were thus prompted to investigate the reaction of the dianionic *N*-*p*-tolyl-fullerooxazolidinone with BnBr to better understand the electrochemical reactivity of heterocyclic fullerene derivatives fused with two different heteroatoms and to obtain novel fullerene derivatives. To our surprise and satisfaction, a ring-opened decarboxylative mono-benzylated 1,4-adduct and bis-benzylated 1,2,3,16- and 1,4,9,25-adducts by dual benzylations accompanied by the heterocycle rearrangement from a [6,6]-bond to a [5,6]-bond could be obtained from the dianionic *N*-*p*-tolyl-fullerooxazolidinone. The phenomenon of ring opening of dianionic C_60_-fused heterocycles followed by decarboxylation from the resulted dianionic species has no precedent in fullerene chemistry. In this article, we describe the details of these electrochemical benzylations as well as the electrochemical properties of the obtained products.

## 2. Results and Discussion

**Figure 1:**
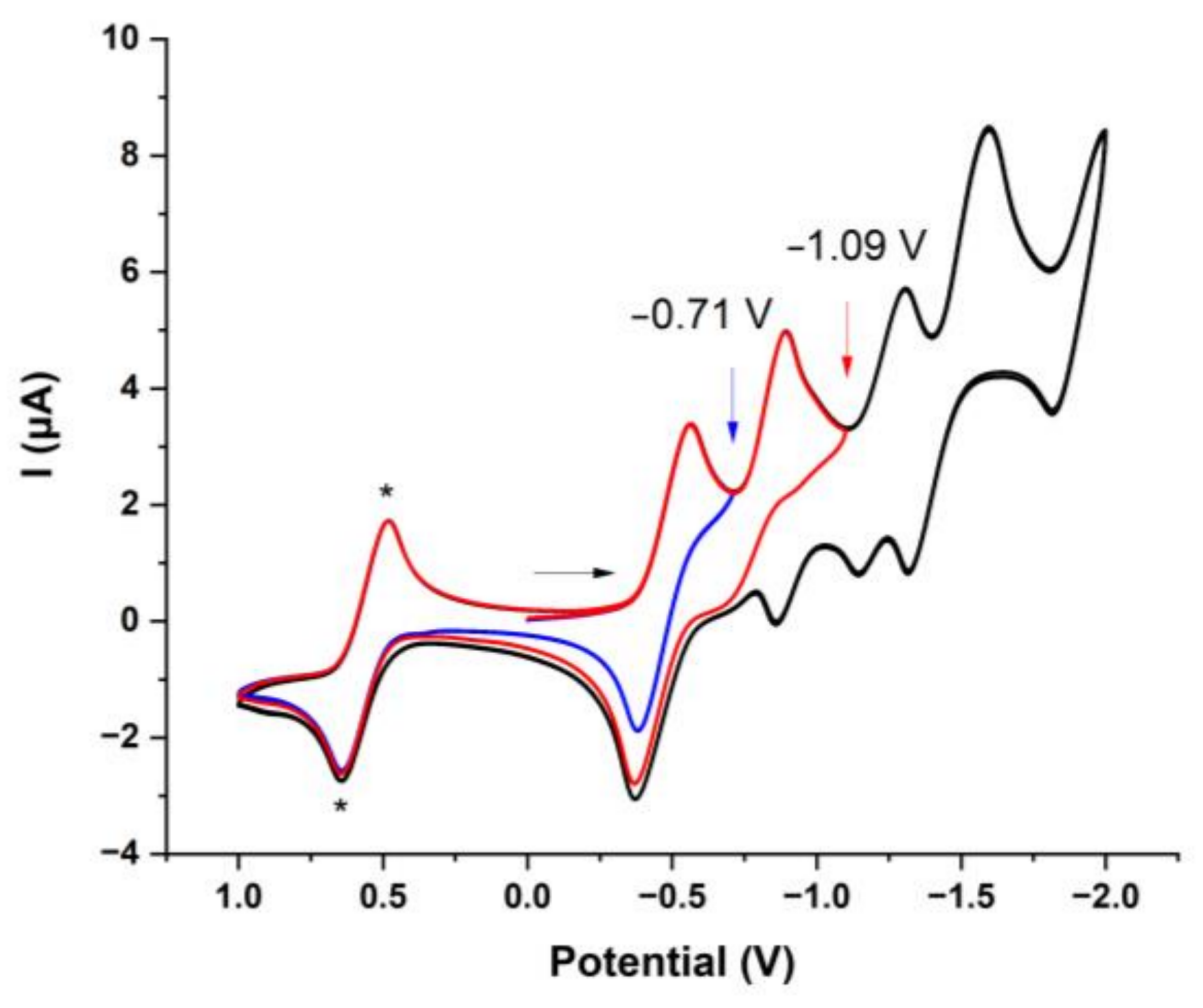
Cyclic voltammogram of **1** (1.0 mM) recorded in 1,2-C_6_H_4_Cl_2_ containing 0.1 M TBAP with ferrocene (1.0 mM) as reference. The parameters of CV: scan rate: 50 mV s^−1^; initial potential: 0.0 V; initial scan polarity: negative. The asterisks * label the ferrocene/ferrocenium.

**Scheme 1:**
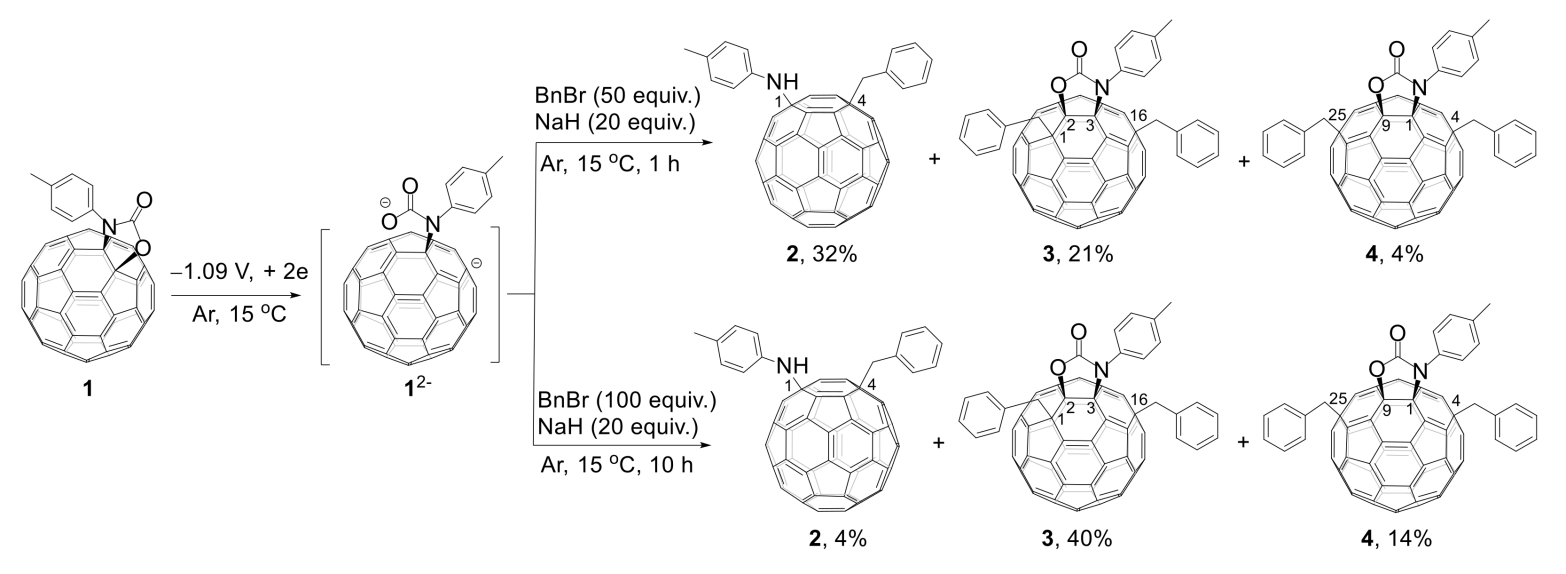
Reaction of the electrochemically generated **1**^2−^ with BnBr under different conditions.

Compound **1** was synthesized by the previously reported procedure [39]. As seen from the cyclic voltammogram (CV) of **1**, the first redox was reversible, whereas the second redox was electrochemically irreversible (Figure 1). It was found that the CV of **1** was essentially the same at 25 °C and a slightly lower temperature such as 15 °C. This result indicated a cleavage of carbon-heteroatom bond of **1** after accepting two electrons. Previous work on fulleroimidazoline and fullerooxazoline derivatives showed that the C_60_–O and C_60_–N could be readily cleaved when they received two electrons [36,37], and we found that **1** (0.02 mmol) could be electrochemically converted at −1.09 V (the trough of the second wave) to dianion **1**^2−^, which was readily decomposed to C_60_ during electrolysis at 25 °C. The electrolysis at lower temperature would slow down the decomposition of dianion **1**^2−^ to C_60_, yet required a longer time to reach the theoretical number and would also cause partial decomposition of dianion **1**^2−^. On balance, we carried out our experiments on **1** (0.01 mmol) at a lower temperature of 15 °C. By controlled potential electrolysis (CPE) of **1** at −1.09 V in 1,2-C_6_H_4_Cl_2_ solution containing 0.1 M tetra-*n*-butylammonium perchlorate (TBAP) at 15 °C under an atmosphere of argon, dianion **1**^2−^ was generated after acceptance of two electrons. Similar to other dianionic C_60_-fused heterocycles, the ring-opened singly-bonded fullerenyl dianion was attained after addition of two electrons [26,27,28,29,30,31,32,33,34,35,36,37], as confirmed by the very different CV of dianion **1**^2−^ (Figure S21) compared to that of neutral **1** (Figure 1). Then, **1**^2−^ was treated with 20 equiv. of NaH and 50 equiv. of BnBr at 15 °C for 1 h (Scheme 1). Surprisingly, a ring-opened mono-benzylated 1,4-adduct **2** was isolated as the major product in 32% yield together with bis-benzylated 1,2,3,16-adduct **3** and 1,4,9,25-adduct **4** in 21% and 4% yields, respectively. The formation of product **2** resulted from decarboxylative benzylation of **1**^2−^, which was in sharp contrast with the previously reported benzylation of dianionic C_60_-fused lactones [40]. The isolation of products **3** and **4** indicated that **1**^2−^ could react with two molecules of BnBr. For the purpose of enhancing the possibility of adding the second BnBr, the amount of BnBr was increased to 100 equiv., and the yield of the mono-benzylated 1,4-adduct **2** was decreased to 23%, while the competing bis-benzylated 1,2,3,16-adduct **3** was obtained in 29% yield as the major product along with 1,4,9,25-adduct **4** in 3% yield as the minor product. In order to obtain more bis-benzylated 1,2,3,16-adduct **3,** the reaction time was further increased to 10 h. Intriguingly, the bis-benzylated 1,2,3,16-adduct **3** could be obtained in 40% yield as the major product along with another bis-benzylated 1,4,9,25-adduct **4** in 14% yield and 1,4-adduct **2** in 4% yield as the minor products. Besides the above products, the reaction mixtures also contained decomposed C_60_, unreacted **1** and insoluble material (see details in Materials and Methods). It seemed that 1,4-adduct **2** was unstable under our experimental conditions, likely due to the attached arylamino moiety. In brief, when the dianion **1**^2−^ was treated with 50 equiv. of BnBr for a short reaction time (1 h), mono-benzylated product **2** was the major product along with bis-benzylated products **3** and **4** as minor products. When the amount of BnBr was increased to 100 equiv. for the purpose that the second BnBr could participate in the reaction system, and reaction time was further prolonged (10 h), products **3** and **4** were obtained exclusively accompanied with a small amount of **2**. As seen from Scheme 1, the product distribution for the benzylation of **1**^2−^ could be dramatically manipulated by varying the amount of BnBr and reaction time, either mono-benzylated 1,4-adduct **2** or bis-benzylated 1,2,3,16-adduct **3** could be obtained as the major product.

Products **2**–**4** were characterized by various spectroscopic data, and **3** was further established by single-crystal X-ray crystallography. The MALDI-TOF mass spectra of **2**–**4** showed the expected molecular ion peaks. In its ^1^H NMR spectrum of **2**, two doublet peaks at 4.07 and 4.03 ppm with a coupling constant of 13.0 Hz for the methylene protons of the benzyl group attached to the fullerene skeleton and a singlet peak at 2.32 ppm for the methyl group of the *p*-toluidine moiety were observed. The ^1^H NMR spectrum of **3** showed an AB quartet at 4.41 and 4.24 ppm with a coupling constant of 13.3 Hz for the methylene protons, while another AB quartet at 2.59 and 2.44 ppm with a coupling constant of 12.9 Hz, indicating that the latter methylene protons were located at the shielding region of a phenyl ring. Likewise, the ^1^H NMR spectrum of **4** displayed the same phenomena for the chemical shifts and splitting patterns of the methylene protons attached to the fullerene skeleton. Herein, the two benzyl groups should be located at the different sides of the oxazolidinone moiety in compounds **3** and **4**. The ^13^C NMR spectrum of **2** showed 50 peaks for the 58 sp^2^-carbon atoms in the 156.15–137.93 ppm range and two sp^3^-carbon atom peaks of the fullerene cage at 68.28 and 60.01 ppm, and the ^13^C NMR spectra of **3** and **4** displayed at least 52 peaks between 156.04–135.95 ppm for the 56 sp^2^-carbon atoms and four peaks between 90.31–56.52 ppm for the four sp^3^-carbon atoms of the fullerene skeleton, agreeing with their *C*_1_ symmetry. The UV-vis spectrum of **2** showed a broad absorption band at 446 nm, which resembled those of other 1,4-adducts of C_60_ [35,40,41]. The UV-vis spectrum of **3** exhibited two strong absorption bands at 254 and 324 nm, together with three weak absorption bands at 405, 431 and 690 nm, which were similar to those of 1,2,3,16-adducts in the literature [26,27,30,31,32,33,35,37,38]. However, in the UV-vis spectrum of product **4**, three strong absorption bands at 250, 268 and 330 nm, together with five weak absorption bands at 409, 450, 510, 591 and 672 nm were observed, and were very similar to those of 1,4,9,25-adducts in the literature [32,35].

**Figure 2:**
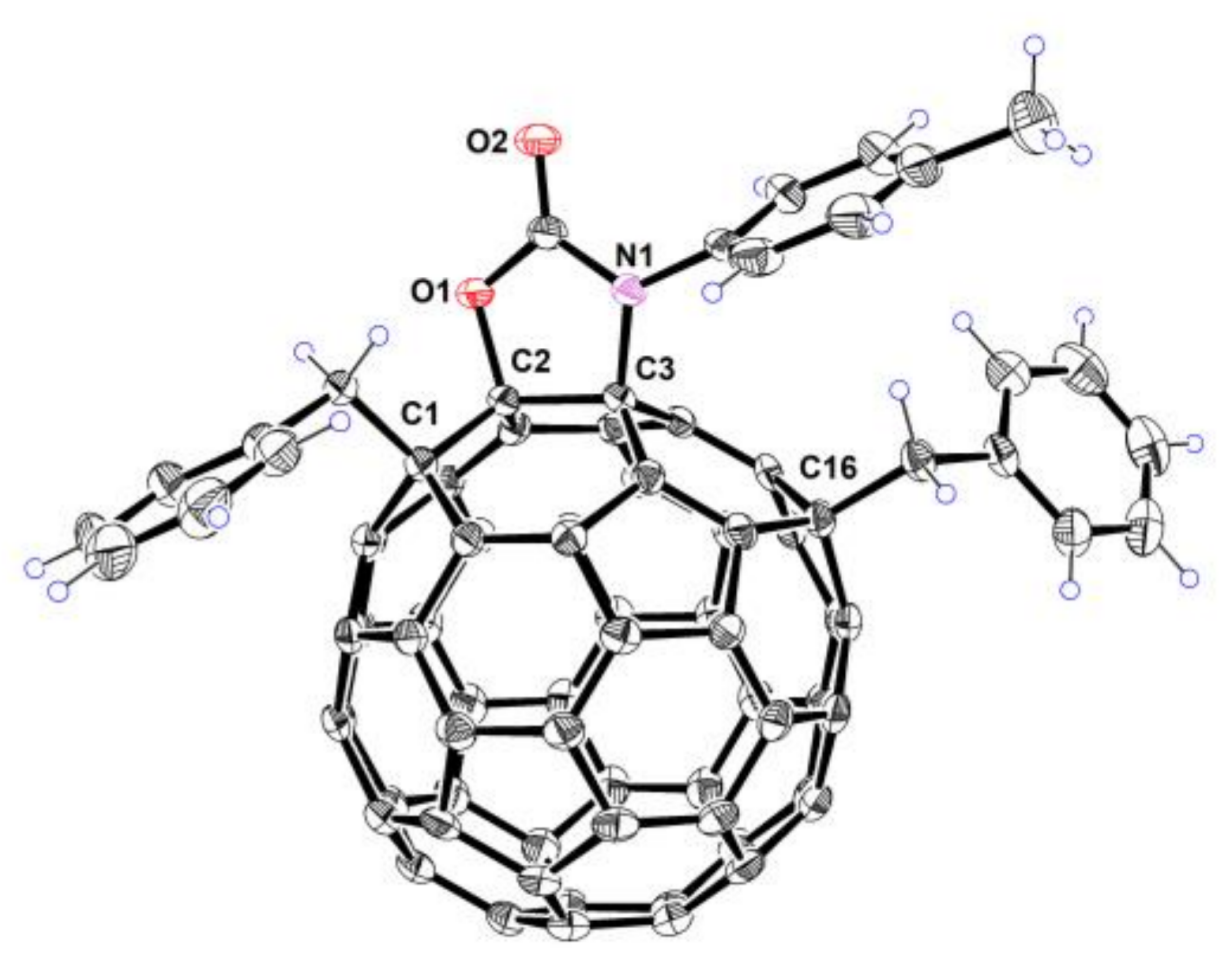
ORTEP diagram of **3** with 30% thermal ellipsoids. The solvent CS_2_ molecule is omitted for clarity.

The single crystal of **3** was obtained by slow evaporation of its carbon disulfide (CS_2_) solution at room temperature. The crystal structure unambiguously demonstrated that the oxazolidinone moiety was located at a [5,6]-bond (C2–C3) of the C_60_ skeleton (Figure 2). One of the benzyl groups was connected to C1, and the other one was attached to the C16 position and was distant from other three addend sites. Due to their sp^3^ character, the four functionalized carbon atoms (C1, C2, C3 and C16) of the fullerene skeleton were uplifted from the spherical surface notably. The single-crystal structure clearly established that the molecular structure of product **3** possessed a 1,2,3,16-addition pattern. Disappointingly, the attempts to obtain the single crystals of **2** and **4** failed. According to the previous literature [26,27,28,29,30,31,32,33,35], the carbon-heteroatom bond of C_60_-fused heterocycles would break after accepting two electrons, and the most negatively charged carbon atom among the non-functionalized carbons of the fullerene cage was resided at the *para* position of the addend, which was the most likely site to react with electrophile. As seen from the structure of **3**, the C16 was located at the *para* position of C3. Therefore, electroreduction led to the breakage of the C−O bond followed by rearrangement of the heterocycle moiety from a [6,6]-bond to a [5,6]-bond on the fullerene cage.

**Scheme 2:**
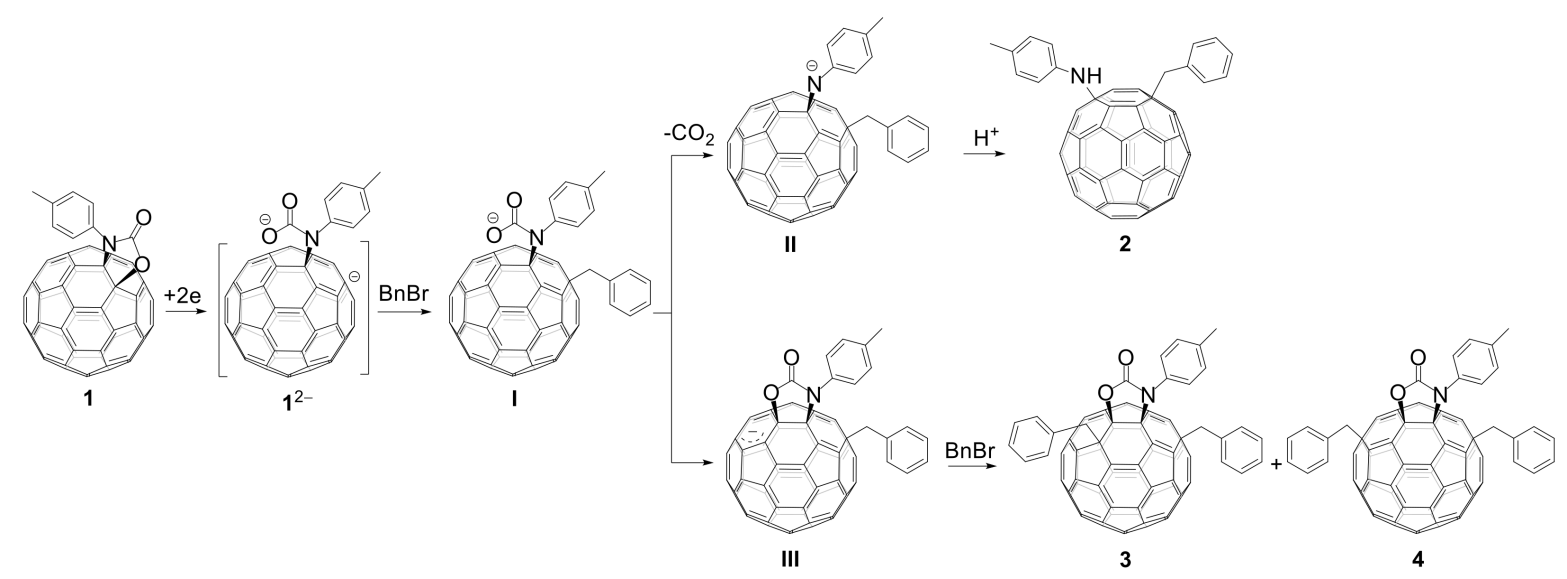
Proposed mechanism for the formations of **2**, **3,** and **4**.

Based on the previous literature [26,27,28,29,30,31,32,33,35,42], a possible mechanism for the formations of **2**, **3,** and **4** from the reaction of **1**^2–^ with BnBr is shown in Scheme 2. After acceptance of two electrons, fullerooxazolidinone **1** is reduced by CPE and undergoes C_60_–O bond breakage to afford the ring-opened dianion **1**^2−^, with one negative charge located at the carbamic oxygen atom and another charge mainly distributed on the *para* position of the carbon attached with the addend, which is prone to react with BnBr to generate intermediate **I** [26,27,28,29,30,31,32,33,35]. Subsequently, the intermediate **I** can proceed via either decarboxylation process to afford **II** [42] or ring-closing process to the adjacent [5,6]-bond to generate **III** [26,27,28,29,30,31,32,33,35]. Then, a protonation process of **II** affords **2**, while **III** can react with the second BnBr to give 1,2,3,16-adduct **3** and 1,4,9,25-adduct **4** controlled by the electronic and steric effects.

**Figure 3:**
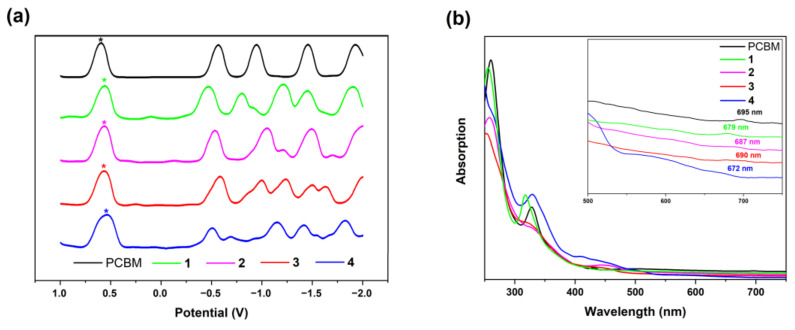
(**a**) DPVs of **1**–**4** along with PCBM recorded in 1,2-C_6_H_4_Cl_2_ containing 0.1 M TBAP. The asterisks label the ferrocene/ferrocenium. (**b**) UV-vis absorption spectra of **1**–**4** along with PCBM in CHCl_3_. The inset displays the expanded range of 500–750 nm. All curves of DPVs and UV-vis absorption spectra are shifted vertically for clarity.

Suitable energy band alignment facilitates charge transport from the perovskite film to electrodes in PSC devices [43]. [6,6]-Phenyl-C_61_-butyric acid methyl ester (PCBM) is a fullerene derivative that has been widely applied in PSC devices [7,8,9,10,11,43]. Therefore, the energy levels of the lowest unoccupied molecular orbital (LUMO) and the highest occupied molecular orbital (HOMO) of products **1**–**4** along with PCBM were obtained through differential pulse voltammetry (DPV) and CV (Figure 1, Figure 3a and Figures S22–S25) combined with UV-vis absorption spectroscopy (Figure 3b). The DPV studies revealed that the first reduction potentials (*E*_1_) of **1**, **2**, **3,** and **4** were −1.028, −1.096, −1.148 and −1.051 V vs. Fc/Fc^+^, respectively. Hence, the LUMO energy levels of **1**, **2**, **3,** and **4** were calculated to be −3.772, −3.704, −3.652 and −3.749 eV, respectively [44,45] (Table 1). According to the optical bandgaps correlated with the absorption onsets in the UV-vis spectra at 679, 687, 690 and 672 nm for **2**, **3** and **4**, the corresponding HOMO energy levels of **1**, **2**, **3,** and **4** were calculated to be −5.598, −5.509, −5.449 and −5.594 eV, respectively [44,46,47]. It could be found that the energy levels of **1**, **2**, **3,** and **4** were also comparable to those of PCBM, indicating that they are promising materials in solar cell devices [7, 8, 9, 10, 11].

## 3. Materials and Methods

### 3.1. General Information

All electrochemical measurements and reactions were performed under an atmosphere of argon atmosphere using a Shanghai Chenhua CHI620E workstation. The electrolyte TBAP was recrystallized from absolute ethanol and then dried in a vacuum at 40 °C prior to use. Other chemicals were obtained commercially and used without further purification. CV measurements were performed in a conventional three-electrode cell. A 2 mm diameter platinum disc electrode and a platinum wire auxiliary electrode were used as working and counter electrodes, respectively, and the reference electrode was saturated calomel electrode (SCE). CPE was carried out on a potentiostat/galvanostat using an “H” type cell. Two platinum gauze (15 mm × 30 mm) electrodes served as working and counter electrodes, respectively, and were separated by a sintered glass frit. The SCE was separated from the bulk of the solution by a fritted-glass bridge of low porosity which contained the solvent/supporting electrolyte mixture.

### 3.2. Generation of Dianion ***1**^2−^*

0.01 mmol of **1** dissolved in 15.0 mL of anhydrous 1,2-C_6_H_4_Cl_2_ solution containing 0.1 M TBAP was added into an “H” type cell. After bubbling with argon for 30 min at 15 °C, the solution was electroreduced by CPE at –1.09 V. When the theoretical number of coulombs (1.93 C) was reached, the electrolysis was terminated after about 1 h, and a dark-green solution of **1**^2−^ was obtained.

### 3.3. Synthesis of Compounds ***2**–**4***

Synthesis of compounds **2**, **3,** and **4** with 50 equiv. of BnBr: The dianion **1**^2−^ was obtained by electroduction from *N*-*p*-tolyl-fullerooxazolidinone **1** (8.7 mg, 0.01 mmol), and then reacted with NaH (57–63% oil dispersion, 8.0 mg, 0.20 mmol) and BnBr (60.0 μL, 0.50 mmol). After the solution was kept stirring at 15 °C for 1 h, the resulting mixture was directly filtered through a silica gel (200–300 mesh) plug with CS_2_/CH_2_Cl_2_ (1:1 *v*/*v*) to remove TBAP and insoluble material. Then, the solvents were removed with a rotary evaporator in vacuo. Next, the residue combined from the two runs was further separated on a silica gel column (300–400 mesh) with CS_2_/CH_2_Cl_2_ (4:1 *v*/*v*) as the eluent to afford compound **2** (5.8 mg, 32%), compound **3** (4.5 mg, 21%), and compound **4** (0.8 mg, 4%) along with unreacted **1** (2.4 mg, 14%) and decomposed C_60_ (2.6 mg, 18%).

Synthesis of compounds **2**, **3,** and **4** with 100 equiv. of BnBr: The dianion **1**^2−^ was obtained by electroduction from *N*-*p*-tolyl-fullerooxazolidinone **1** (8.7 mg, 0.01 mmol), and then reacted with NaH (57–63% oil dispersion, 8.0 mg, 0.20 mmol) and BnBr (120.0 μL, 1.00 mmol). After the solution was kept stirring at 15 °C for 1 h, the resulting mixture was directly filtered through a silica gel (200–300 mesh) plug with CS_2_/CH_2_Cl_2_ (1:1 *v*/*v*) to remove TBAP and insoluble materials, and then removed the solvent with a rotary evaporator in vacuo. Next, the residue combined from the two runs was further separated on a silica gel column (300–400 mesh) with CS_2_/CH_2_Cl_2_ (4:1 *v*/*v*) as the eluent to afford compound **2** (4.2 mg, 23%), compound **3** (6.2 mg, 29%), and compound **4** (0.6 mg, 3%) along with unreacted **1** (1.7 mg, 10%) and decomposed C_60_ (3.0 mg, 21%). When the reaction time was prolonged to 10 h, the same procedure gave compound **2** (0.7 mg, 4%), compound **3** (8.5 mg, 40%), and compound **4** (2.9 mg, 14%) along with decomposed C_60_ (3.1 mg, 22%).

### 3.4. Single-Crystal Growth and Characterization of ***3***

Black block crystals of **3** were obtained by slow evaporation of the CS_2_ solution of **3** at room temperature. Single-crystal X-ray diffraction data were collected on a diffractometer (SuperNova, Rigaku, Rigaku Polska Sp. Z oo UI, Wroclaw, Poland) equipped with a CCD area detector using graphite-monochromated Cu Kα radiation (λ = 1.54184 Å) in the scan range 8.174° < 2θ < 150.64°. Using Olex2, the structure was solved with the ShelXT structure solution program using Intrinsic Phasing and refined with the ShelXL refinement package using Least Squares minimization. Crystallographic data have been deposited in the Cambridge Crystallographic Data Centre as deposition number CCDC 2163434.

### 3.5. CVs and DPVs of Compounds ***1**–**4*** along with PCBM

Compounds **1**–**4** or PCBM (1.0 mM) dissolved in anhydrous 1,2-C_6_H_4_Cl_2_ solution containing 0.1 M TBAP and ferrocene (1.0 mM) as reference were added into an electrochemical cell under an argon atmosphere at 25 °C, while the CV measurements of **1** (1.0 mM) and dianion **1**^2−^ at 15 °C. CV measurements were then undertaken at a scan rate of 50 mV s^−1^. The parameters of DPV: step potential: 4 mV; pulse amplitude: 50 mV; pulse duration: 0.05 s; pulse period: 0.5 s.

## 4. Conclusions

In conclusion, benzylation of the electrochemically generated dianionic *N*-*p*-tolyl-fullerooxazolidinone with benzyl bromide affords a ring-opened mono-benzylated 1,4-adduct **2**, bis-benzylated 1,2,3,16-adduct **3** and 1,4,9,25-adduct **4**. The product distribution can be manipulated by altering the reaction conditions. The structural assignments for products **2**–**4** are established by spectroscopic data and single-crystal X-ray crystallography. A possible reaction mechanism for the formation of the three types of adducts is proposed. The present work provides a new avenue for the electrochemical functionalization of C_60_-fused heterocycles.

## Figures and Tables

**Table 1:** Characteristic potential values and energy levels of compounds **1**–**4** along with PCBM derived from DPV and UV-vis absorption spectroscopy.

Compd	*E*_1_*^a^* (V)	λ_onset_ *^b^* (nm)	*E*_g,opt_*^c^* (eV)	LUMO Level *^d^* (eV)	HOMO Level *^e^* (eV)
**1**	−1.028	679	1.826	−3.772	−5.598
**2**	−1.096	687	1.805	−3.704	−5.509
**3**	−1.148	690	1.797	−3.652	−5.449
**4**	−1.051	672	1.845	−3.749	−5.594
PCBM	−1.164	695	1.784	−3.636	−5.420

*^a^* Versus ferrocene/ferrocenium. *^b^* Attained from UV-vis spectra. *^c^ E*_g,opt_ = 1240/λ_onset_ [44,47]. *^d^* Estimated using the following equation: LUMO level = −(4.8 + *E*_1_) eV [44,45]. *^e^* HOMO level = (LUMO − *E*_g,opt_) eV [44,46].

## Data Availability

The data presented in this study are available in the article and Supplementary Materials.

## Supplementary Materials

The following supporting information can be downloaded at: https://www.mdpi.com/article/10.3390/nano12132281/s1, General information; Characterization of compounds **2**–**4**; Figure S1. ^1^H NMR (400 MHz, CS_2_ with DMSO-*d*_6_ as the external deuterium lock) of compound **2**; Figure S2. ^13^C NMR (101 MHz, 1:1 CS_2_/CDCl_3_) of compound **2**; Figure S3. Expanded ^13^C NMR (101 MHz, 1:1 CS_2_/CDCl_3_) of compound **2**; Figure S4. Expanded ^13^C NMR (101 MHz, 1:1 CS_2_/CDCl_3_) of compound **2**; Figure S5. Expanded ^13^C NMR (101 MHz, 1:1 CS_2_/CDCl_3_) of compound **2**; Figure S6. ^1^H NMR (400 MHz, CS_2_ with DMSO-*d*_6_ as the external deuterium lock) of compound **3**; Figure S7. ^13^C NMR (101 MHz, CS_2_ with DMSO-*d*_6_ as the external deuterium lock) of compound **3**; Figure S8. Expanded ^13^C NMR (101 MHz, CS_2_ with DMSO-*d*_6_ as the external deuterium lock) of compound **3**; Figure S9. Expanded ^13^C NMR (101 MHz, CS_2_ with DMSO-*d*_6_ as the external deuterium lock) of compound **3**; Figure S10. Expanded ^13^C NMR (101 MHz, CS_2_ with DMSO-*d*_6_ as the external deuterium lock) of compound **3**; Figure S11. ^1^H NMR (400 MHz, CS_2_ with DMSO-*d*_6_ as the external deuterium lock) of compound **4**; Figure S12. ^13^C NMR (101 MHz, CS_2_ with DMSO-*d*_6_ as the external deuterium lock) of compound **4**; Figure S13. Expanded ^13^C NMR (101 MHz, CS_2_ with DMSO-*d*_6_ as the external deuterium lock) of compound **4**; Figure S14. Expanded ^13^C NMR (101 MHz, CS_2_ with DMSO-*d*_6_ as the external deuterium lock) of compound **4**; Figure S15. Expanded ^13^C NMR (101 MHz, CS_2_ with DMSO-*d*_6_ as the external deuterium lock) of compound **4**; Figure S16. UV-vis spectrum of compound **1** in CHCl_3_; Figure S17. UV-vis spectrum of compound **2** in CHCl_3_; Figure S18. UV-vis spectrum of compound **3** in CHCl_3_; Figure S19. UV-vis spectrum of compound **4** in CHCl_3_; Figure S20. UV-vis spectrum of PCBM in CHCl_3_; Figure S21. CV of dianion **1**^2–^ (1.0 mM) recorded in 1,2-C_6_H_4_Cl_2_ containing 0.1 M TBAP at 15 °C. The parameters of CV: scan rate: 50 mV s^−1^; initial potential: –1.09 V; initial scan polarity: negative or positive; Figure S22. CV of compound **2** (1.0 mM) recorded in 1,2-C_6_H_4_Cl_2_ containing 0.1 M TBAP with ferrocene (1.0 mM) as reference at 25 °C. The parameters of CV: scan rate: 50 mV s^−1^; initial potential: 0.0 V; initial scan polarity: negative. The asterisks label the ferrocene/ferrocenium; Figure S23. CV of compound **3** (1.0 mM) recorded in 1,2-C_6_H_4_Cl_2_ containing 0.1 M TBAP with ferrocene (1.0 mM) as reference at 25 °C. The parameters of CV: scan rate: 50 mV s^−1^; initial potential: 0.0 V; initial scan polarity: negative. The asterisks label the ferrocene/ferrocenium; Figure S24. CV of compound **4** (1.0 mM) recorded in 1,2-C_6_H_4_Cl_2_ containing 0.1 M TBAP with ferrocene (1.0 mM) as reference at 25 °C. The parameters of CV: scan rate: 50 mV s^−1^; initial potential: 0.0 V; initial scan polarity: negative. The asterisks label the ferrocene/ferrocenium; Figure S25. CV of PCBM (1.0 mM) recorded in 1,2-C_6_H_4_Cl_2_ containing 0.1 M TBAP with ferrocene (1.0 mM) as reference at 25 °C. The parameters of CV: scan rate: 50 mV s^−1^; initial potential: 0.0 V; initial scan polarity: negative. The asterisks label the ferrocene/ferrocenium; Table S1. Crystal Data and Structure Refinement for Compound **3.**
