# Supplementary material for: Electrochemically Promoted Benzylation of [60]Fullerooxazolidinone
Source: Nanomaterials (Basel). 2022 Jul 1;12(13):2281. doi: 10.3390/nano12132281 (PMC9268232; doi:10.3390/nano12132281)
Supplement: Supplementary file 1 [file nanomaterials-12-02281-s001.zip › nanomaterials-1730616-supplementary.pdf]

Supplementary Material

Electrochemically Promoted Benzylation of [60]Fullerooxazolidinone

Xing-Xing Yan [1], Chuang Niu [1], Shi-Qi Ye [1], Bo-Chen Zhao [1] and Guan-Wu Wang [1,2,*]

[1] Hefei National Research Center for Physical Sciences at the Microscale and Department of Chemistry, University of Science and Technology of China, Hefei 230026, China; yxx1122@mail.ustc.edu.cn (X.-X.Y.); cniu@mail.ustc.edu.cn (C.N.); yeshiqi@mail.ustc.edu.cn (S.-Q.Y.); zbc1946974933@mail.ustc.edu.cn (B.-C.Z.)
[2] State Key Laboratory of Applied Organic Chemistry, Lanzhou University, Lanzhou 730000, China
* Correspondence: gwang@ustc.edu.cn; Tel.: +86-551-6360-7864

1. General information

NMR spectra were recorded on a 400 MHz NMR spectrometer (400 MHz for ^{1}H NMR; 101MHz for ^{13}C NMR). ^{1}H NMR chemical shifts were determined relative to TMS or residual DMSO-d_6 (δ 2.72 ppm). ^{13}C NMR chemical shifts were determined relative to TMS or residual DMSO-d_6 (δ 39.03 ppm). Data for ^{1}H NMR and ^{13}C NMR are reported as follows: chemical shift (δ, ppm), multiplicity (s = singlet, d = doublet, t = triplet, m = multiplet). High-resolution mass spectra (HRMS) were measured with MALDI-TOF in a negative mode.

2. Characterization of compounds 2–4

Spectral data of **2**: ^{1}H NMR (400 MHz, CS_2 with DMSO-d_6 as the external deuterium lock), δ 7.53 (d, J = 7.2 Hz, 2H), 7.43 (t, J = 7.5 Hz, 2H), 7.33 (t, J = 7.4 Hz, 1H), 7.16 (d, J = 8.2 Hz, 2H), 7.06 (d, J = 8.2 Hz, 2H), 4.07 (d, J = 13.0 Hz, 1H), 4.03 (d, J = 13.0 Hz, 1H), 2.32 (s, 3H); ^{13}C NMR (101 MHz, 1:1 CS_2/$CDCl_3$, all 1C unless indicated) δ 156.15, 152.88, 152.48, 150.29, 149.40, 148.77, 148.48, 147.84, 147.61, 147.08, 147.03, 147.00, 146.96, 146.88, 146.74, 146.67, 146.47, 145.63, 145.54, 145.33, 144.97, 144.81, 144.47 (2C), 144.39, 144.22 (2C), 144.19, 144.13 (2C), 144.09, 144.03, 143.99, 143.92, 143.59, 143.32, 143.28, 143.23 (5C), 142.98, 142.90, 142.73, 142.66, 142.59, 142.51, 142.37, 142.29, 142.03, 141.99 (2C), 141.17, 140.59, 139.39, 138.86, 138.08, 137.93, 136.02 (aryl *C*), 133.11 (aryl *C*), 131.02 (2C, aryl *C*), 129.55 (2C, aryl *C*), 128.74(2C, aryl *C*), 127.82 (aryl *C*), 124.28 (2C, aryl *C*), 68.28 (sp^3-*C* of C_{60}), 60.01 (sp^3-*C* of C_{60}), 48.60, 20.94; UV-vis ($CHCl_3$) λ_{max}/nm (log ε) 258 (4.99), 329 (4.46), 446 (3.78), 540 (3.16), 612 (2.93), 687 (2.54); FT-IR ν/cm^{-1} (KBr) 2922, 2852, 1653, 1616, 1509, 1458, 1429, 1377, 1302, 1245, 1187, 1106, 1030, 804, 756, 731, 723, 699, 569, 527; MALDI-TOF MS m/z calcd for $C_{74}H_{15}N$ $[M]^-$917.1210, found 917.1218.

Spectral data of **3**: ^{1}H NMR (400 MHz, CS_2 with DMSO-d_6 as the external deuterium lock) δ 7.47 (s, 4H), 7.30 (d, J = 6.8 Hz, 2H), 7.21–7.13 (m, 3H), 7.11–7.06 (m, 3H), 6.87–6.82 (m, 2H), 4.41 (d, J = 13.3 Hz, 1H), 4.24 (d, J = 13.3 Hz, 1H), 2.59 (d, J = 12.9 Hz, 1H), 2.44 (d, J = 12.9 Hz, 1H), 2.43 (s, 3H); ^{13}C NMR (101 MHz, CS_2 with DMSO-d_6 as the external deuteriumlock, all 1C unless indicated) δ 157.40 (*C*=O), 155.52, 152.41, 150.28, 149.78, 149.36, 149.07, 148.94, 147.85, 147.49, 147.17, 147.00, 146.96, 146.23, 145.93, 145.88, 145.53, 145.50, 145.37, 145.03 (3C), 144.93, 144.92, 144.79, 144.72, 144.58, 144.28, 144.25, 144.07, 143.81, 143.71, 143.60, 143.33, 143.20, 143.13, 143.09, 142.56, 142.23, 141.68, 141.58, 141.05, 140.64, 140.58, 140.30, 139.91, 139.84, 139.77, 139.74, 139.64, 138.94, 138.86, 138.78 (2C), 137.82, 137.49, 135.95, 134.66 (aryl *C*), 133.99 (aryl *C*), 132.66 (aryl *C*), 131.78 (aryl *C*), 130.79 (2C, aryl *C*), 130.55 (2C, aryl *C*), 129.43 (aryl *C*), 129.25 (2C, aryl *C*), 127.81 (aryl *C*), 127.06 (2C, aryl *C*), 126.89 (2C, aryl *C*), 126.16 (aryl *C*), 126.13 (aryl *C*), 90.31 (sp^3-*C* of C_{60}), 72.32 (sp^3-*C* of C_{60}), 61.43 (sp^3-*C* of C_{60}), 57.40 (sp^3-*C* of C_{60}), 45.61, 45.02, 20.49; UV-vis ($CHCl_3$)

λ_{max}/nm (log ε) 254 (4.95), 324 (4.52), 405 (3.85), 431 (3.77), 690 (2.60); FT-IR ν/cm^{-1} (KBr) 2919, 2850, 1771, 1513, 1494, 1454, 1361, 1232, 1155, 1099, 1024, 1008, 862, 816, 791, 741, 723, 699, 562, 534, 527; MALDI-TOF MS m/z calcd for $C_{82}H_{21}NO_2$ $[M]^-$1051.1578, found 1051.1563.

Spectral data of **4**: ^{1}H NMR (400 MHz, CS_2 with DMSO-d_6 as the external deuterium lock) δ 7.47–7.40 (m, 4H), 7.33–7.23 (m, 5H), 7.13–7.10 (m, 3H), 6.86–6.82 (m, 2H), 3.97 (d, J = 13.0 Hz, 1H), 3.88 (d, J = 13.0 Hz, 1H), 2.54 (d, J = 12.9 Hz, 1H), 2.44 (d, J = 12.9 Hz, 1H), 2.41 (s, 3H); ^{13}C NMR (101 MHz, CS_2 with DMSO-d_6 as the external deuterium lock, all 1C unlessindicated) δ 157.41 (C=O), 156.04, 153.47, 153.41, 152.66, 152.10, 151.46, 150.03, 149.75, 148.80, 148.55, 147.51, 147.37 (2C), 147.01, 146.95, 146.37, 146.28, 146.10, 145.89, 145.87, 145.80, 145.66 (2C), 145.38, 144.90, 144.78, 143.97, 143.90, 143.85, 143.82, 143.76, 143.51, 143.42, 143.27, 143.21, 143.02, 142.92, 142.84, 142.54, 142.44, 141.92, 141.87, 141.74, 141.44, 141.25, 141.13, 140.99 (2C), 139.90, 139.38, 139.11, 138.83 (2C), 138.30, 137.99, 137.08, 133.63(aryl C), 133.55 (aryl C), 131.89 (aryl C), 131.19 (aryl C), 130.44 (2C, aryl C), 129.42 (2C, aryl$C$), 129.40 (2C, aryl C), 129.23 (2C, aryl C), 127.46 (2C, aryl C), 126.99 (2C, aryl C), 126.59 (aryl C), 126.23 (aryl C), 81.13 (sp^3-C of C_{60}), 70.52 (sp^3-C of C_{60}), 57.17 (sp^3-C of C_{60}), 56.52 (sp^3-C of C_{60}), 47.34, 45.90, 20.49; UV-vis ($CHCl_3$) λ_{max}/nm (log ε) 250 (5.08), 268 (4.99), 330 (4.72), 409 (4.12), 450 (3.99), 510 (3.63), 591 (3.20), 672 (2.70); FT-IR ν/cm^{-1} (KBr) 2920, 2851, 1771, 1514, 1494, 1455, 1437, 1358, 1229, 1161, 1129, 1079, 1019, 863, 791, 749, 728, 718, 698, 590, 558, 531; MALDI-TOF MS m/z calcd for $C_{82}H_{21}NO_2$ $[M]^-$1051.1578, found 1051.1575.

3. NMR spectra of compounds 2–4

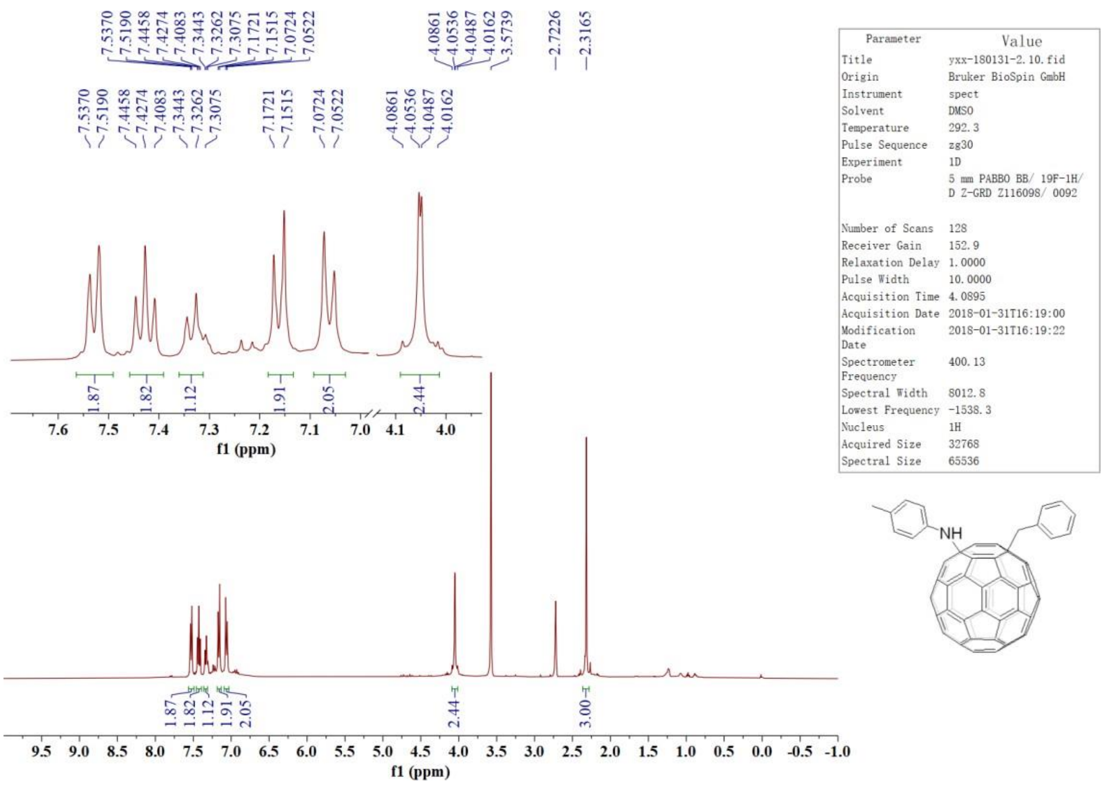

Figure S1. ^{1}H NMR (400 MHz, CS_2 with DMSO-d_6 as the external deuterium lock) of compound **2**.

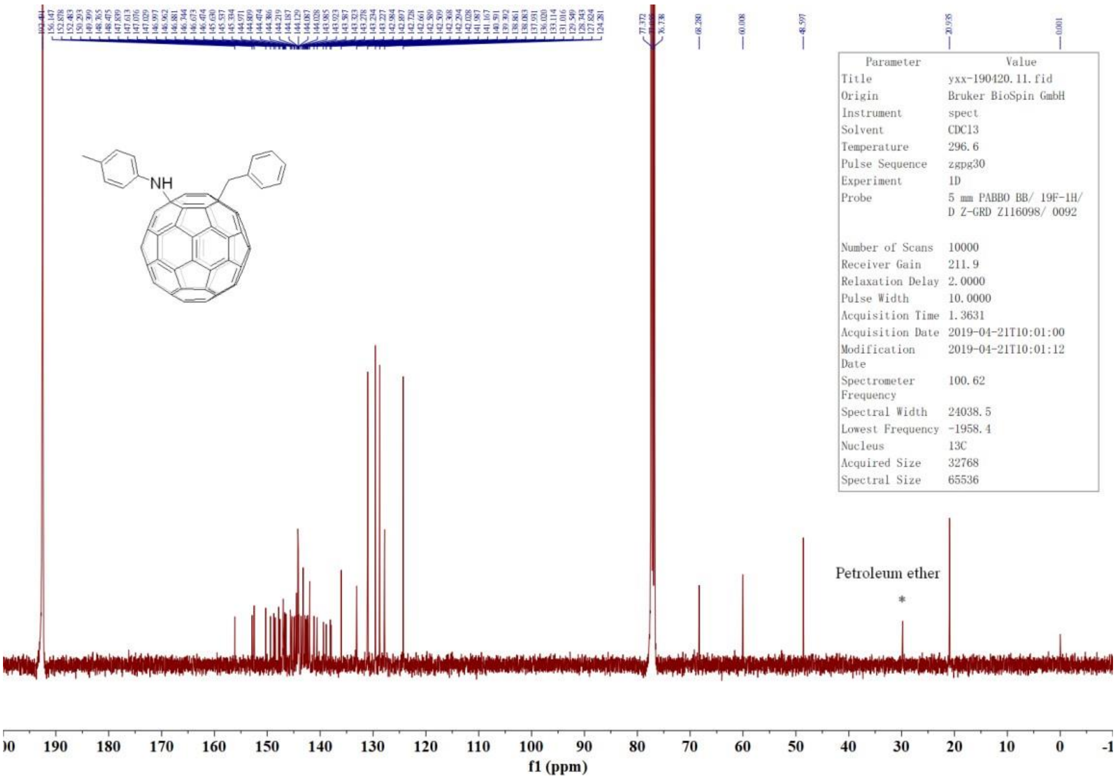

Figure S2. ^{13}C NMR (101 MHz, 1:1 CS_2/$CDCl_3$) of compound **2**.

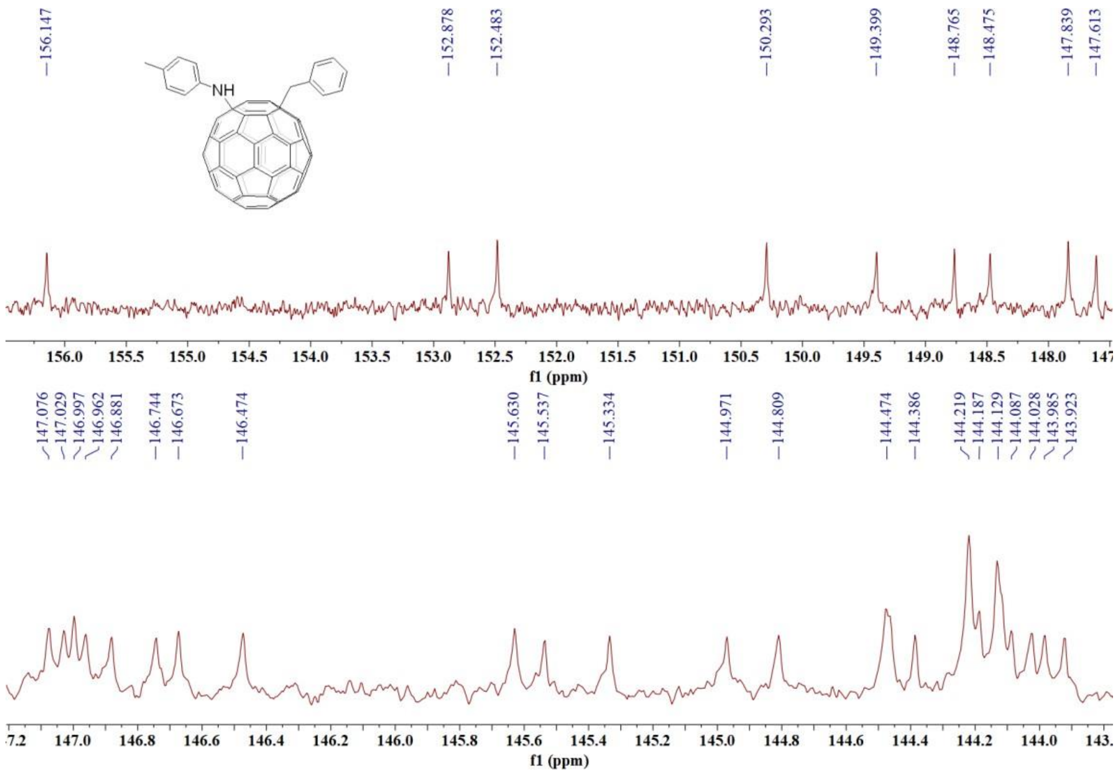

Figure S3. Expanded ^{13}C NMR (101 MHz, 1:1 $CS_2/CDCl_3$) of compound **2**.

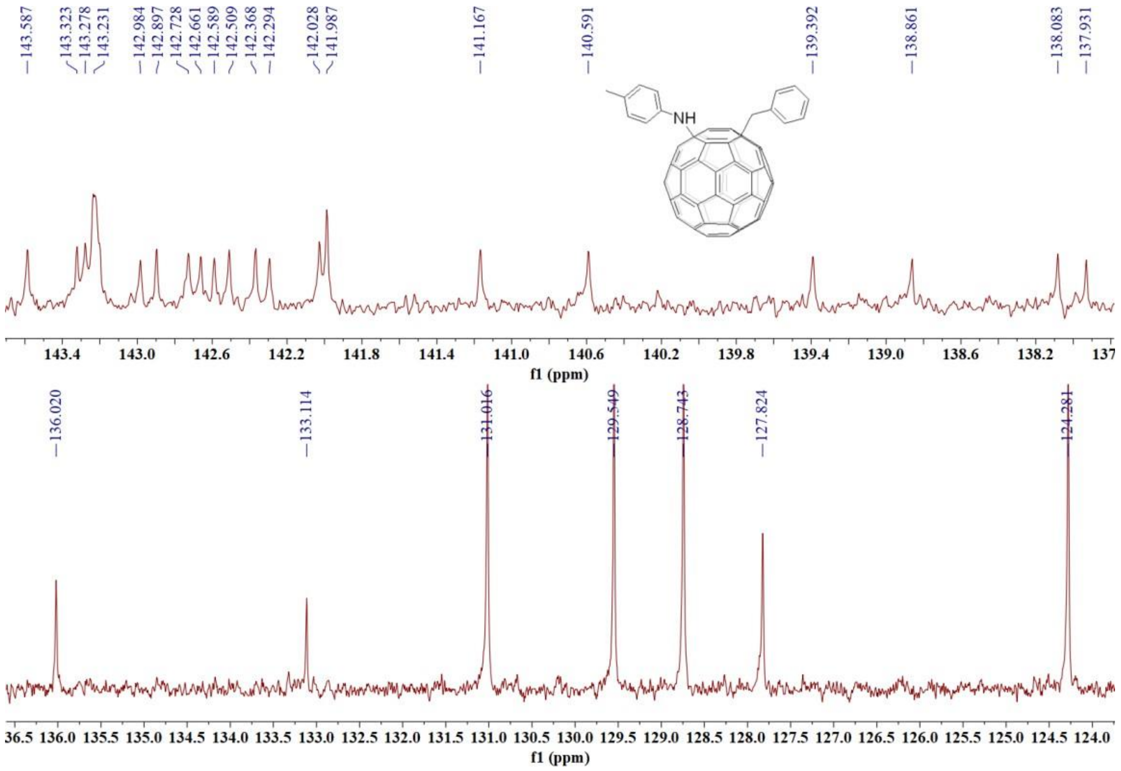

Figure S4. Expanded ^{13}C NMR (101 MHz, 1:1 $CS_2/CDCl_3$) of compound **2**.

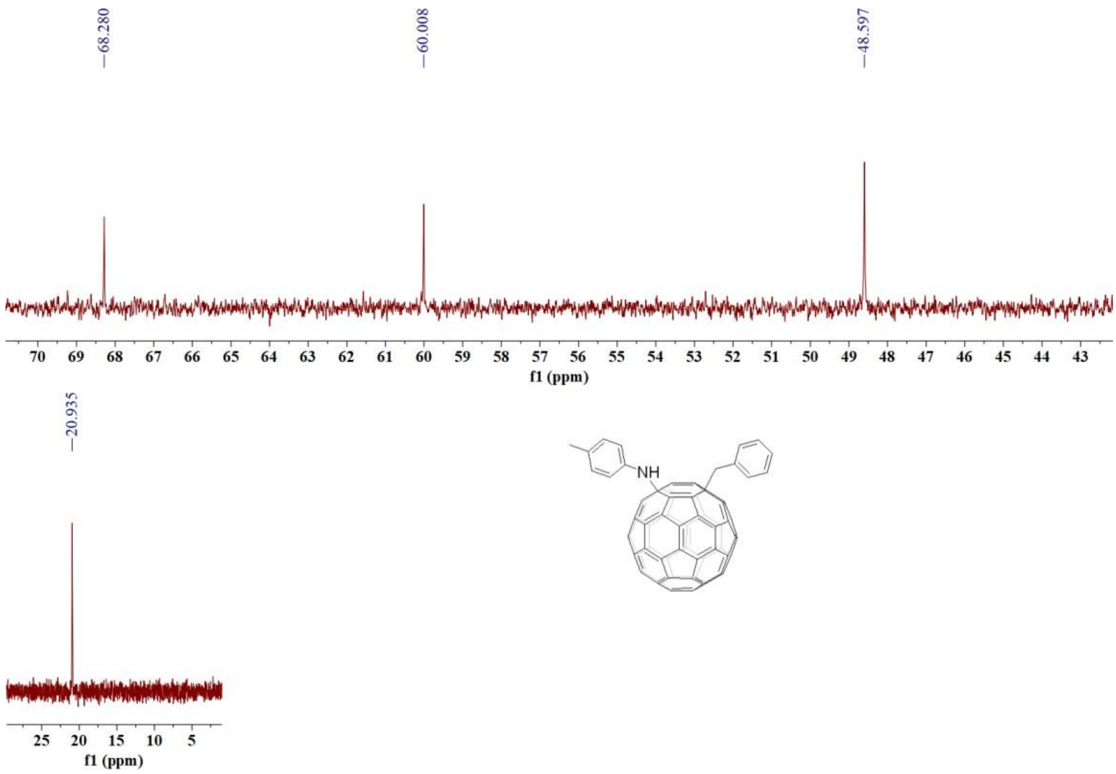

Figure S5. Expanded ^{13}C NMR (101 MHz, 1:1 $CS_2/CDCl_3$) of compound **2**.

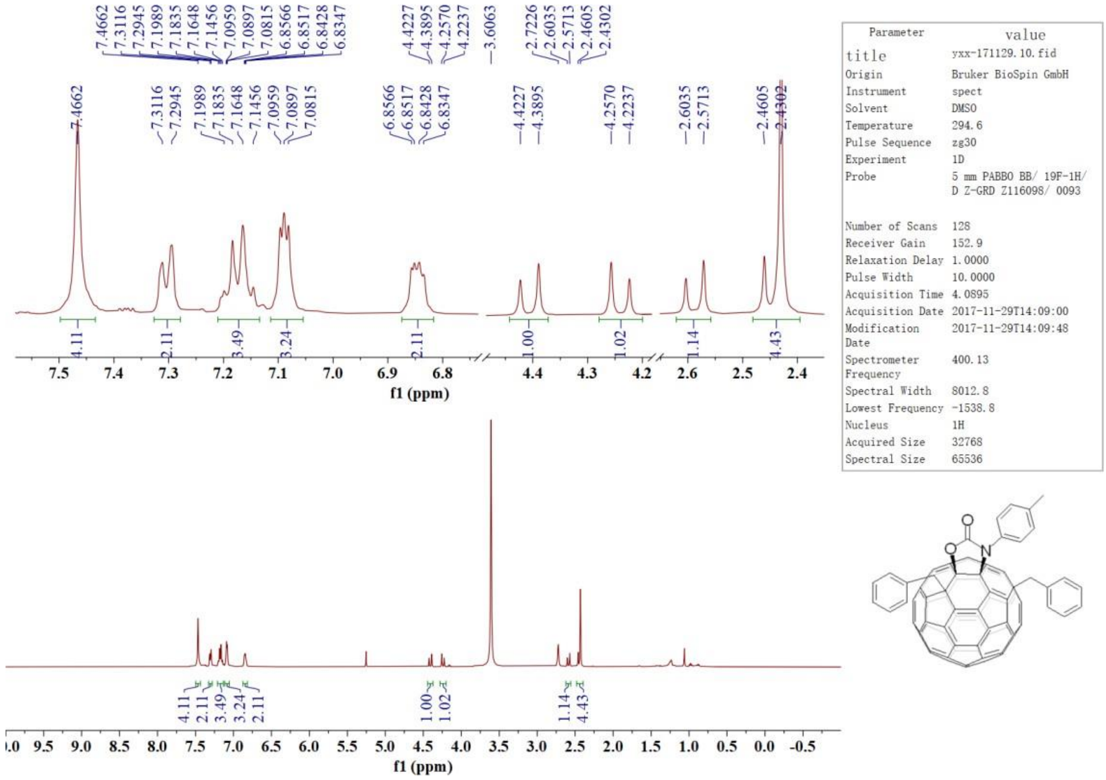

Figure S6. ^{1}H NMR (400 MHz, CS_2 with DMSO-d_6 as the external deuterium lock) of compound **3**.

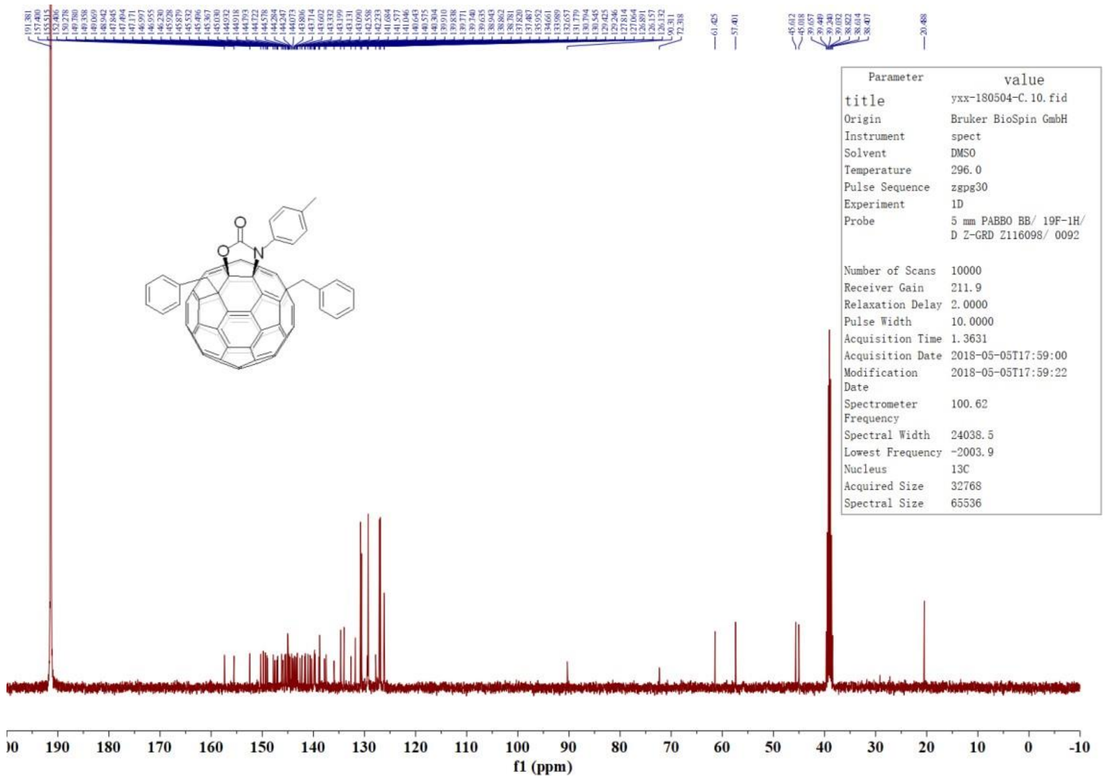

Figure S7. ^{13}C NMR (101 MHz, CS_2 with DMSO-d_6 as the external deuterium lock) of compound **3**.

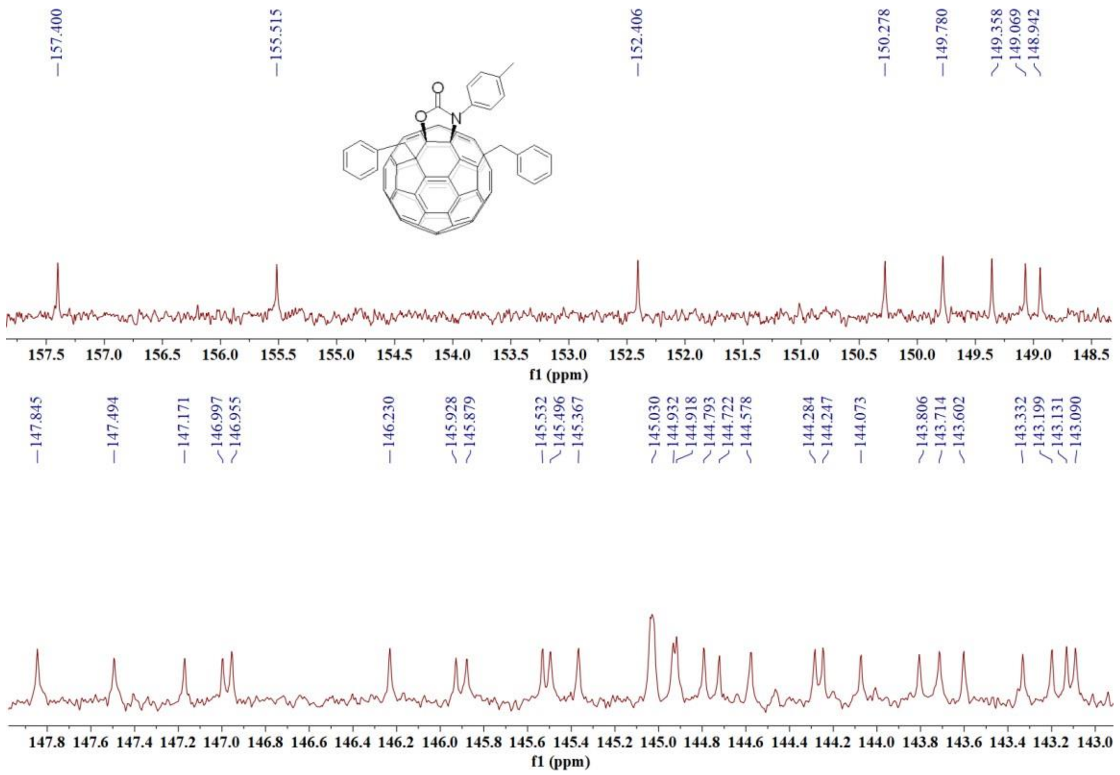

Figure S8. Expanded ^{13}C NMR (101 MHz, CS_2 with DMSO-d_6 as the external deuterium lock) ofcompound **3**.

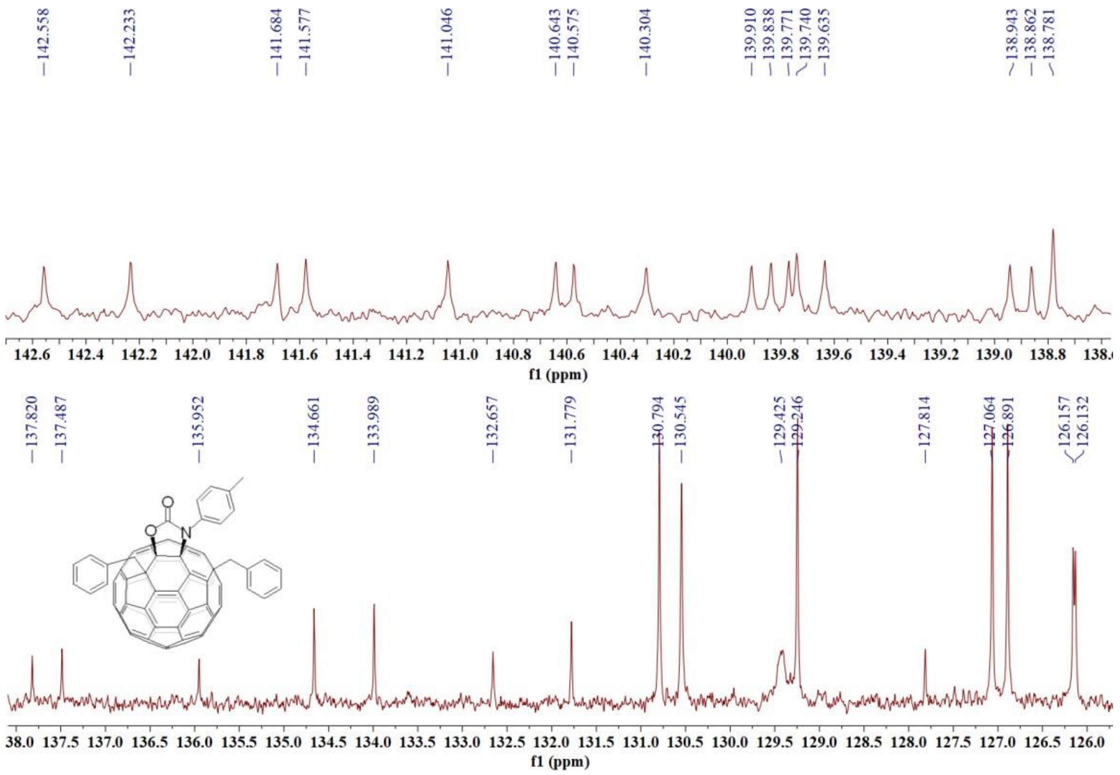

Figure S9. Expanded ^{13}C NMR (101 MHz, CS_2 with DMSO-d_6 as the external deuterium lock) ofcompound **3**.

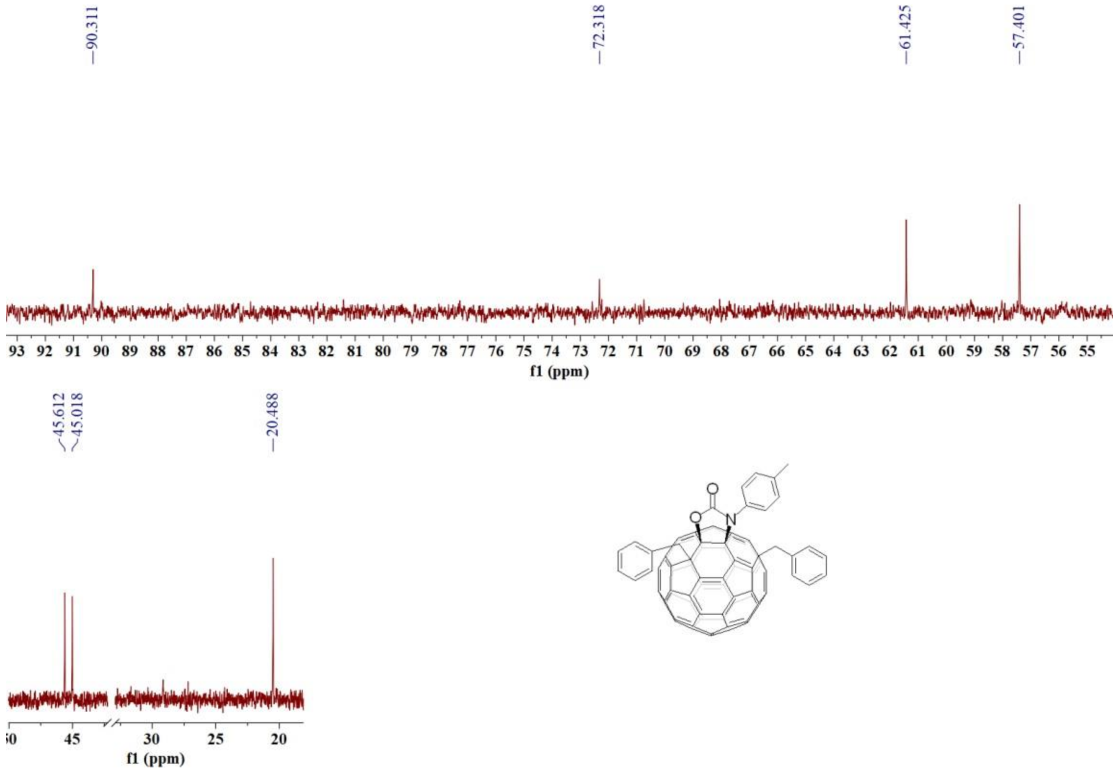

Figure S10. Expanded ^{13}C NMR (101 MHz, CS_2 with DMSO-d_6 as the external deuterium lock) ofcompound **3**.

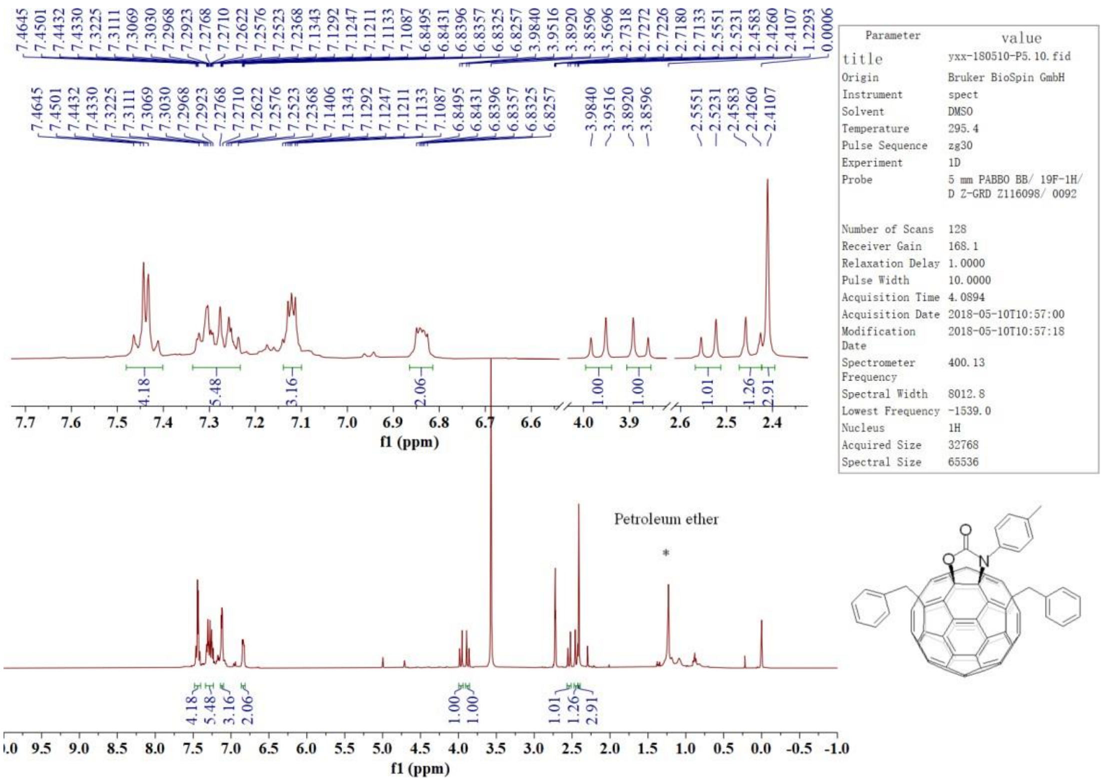

Figure S11. ^{1}H NMR (400 MHz, CS_2 with DMSO-d_6 as the external deuterium lock) of compound **4**.

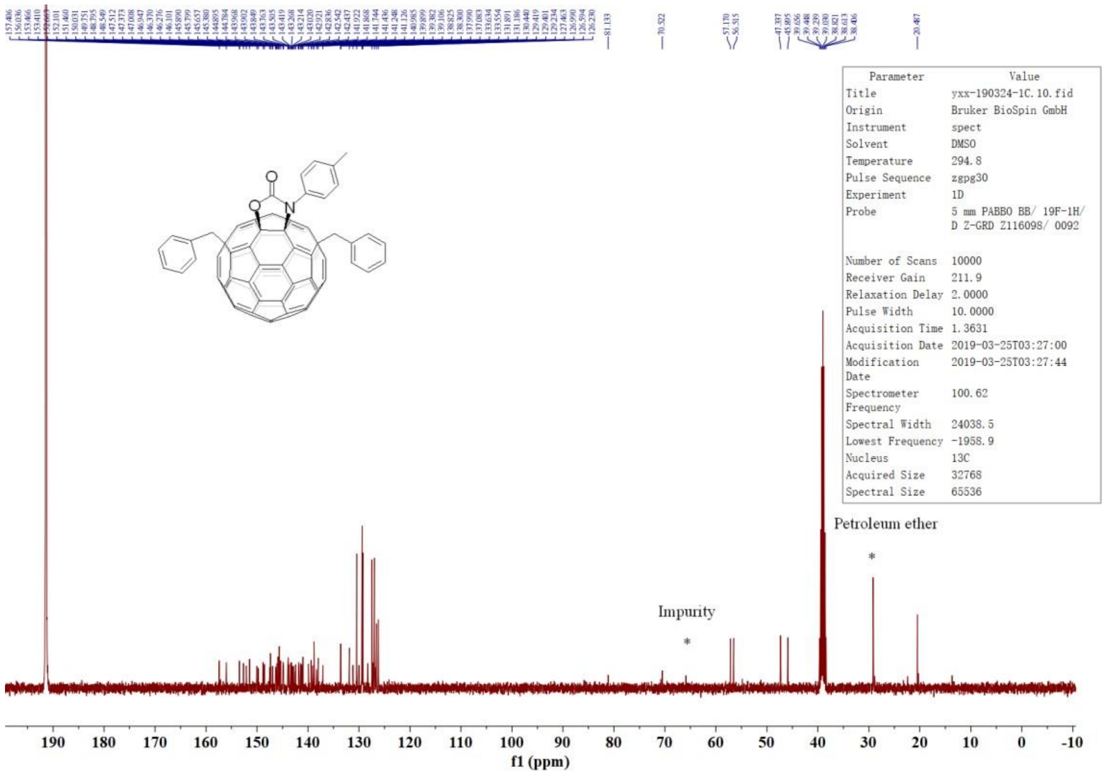

Figure S12. ^{13}C NMR (101 MHz, CS_2 with DMSO-d_6 as the external deuterium lock) of compound **4**.

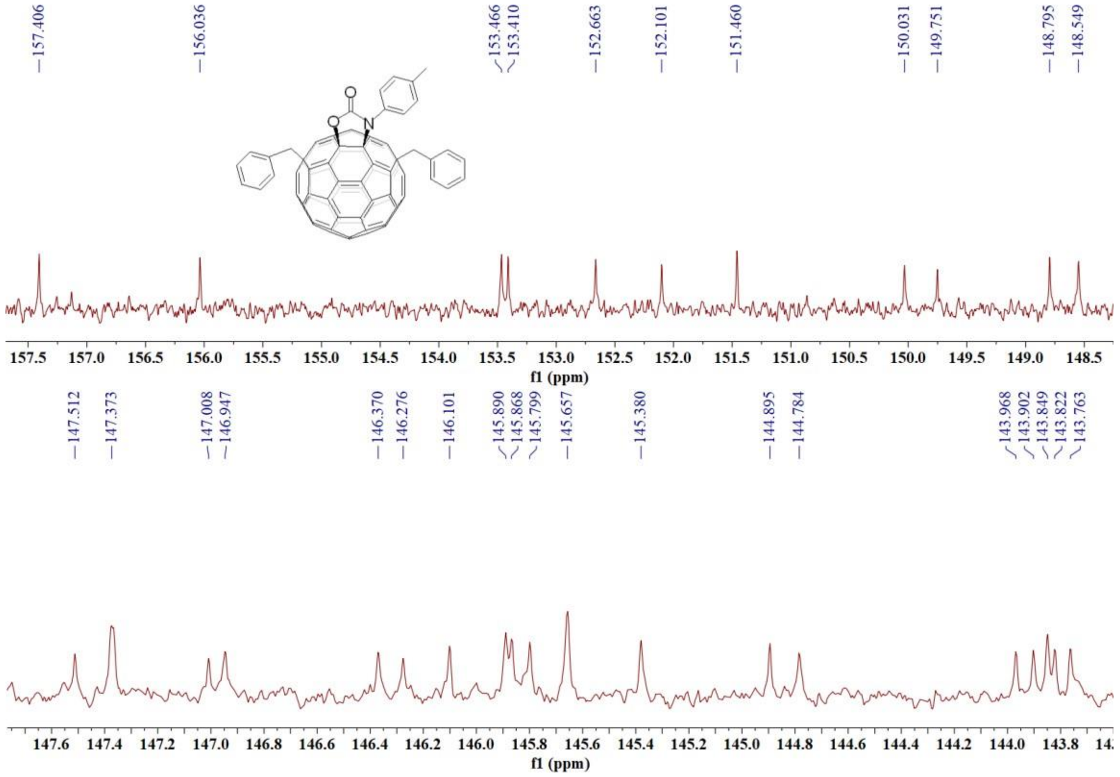

Figure S13. Expanded ^{13}C NMR (101 MHz, CS_2 with DMSO-d_6 as the external deuterium lock) ofcompound **4**.

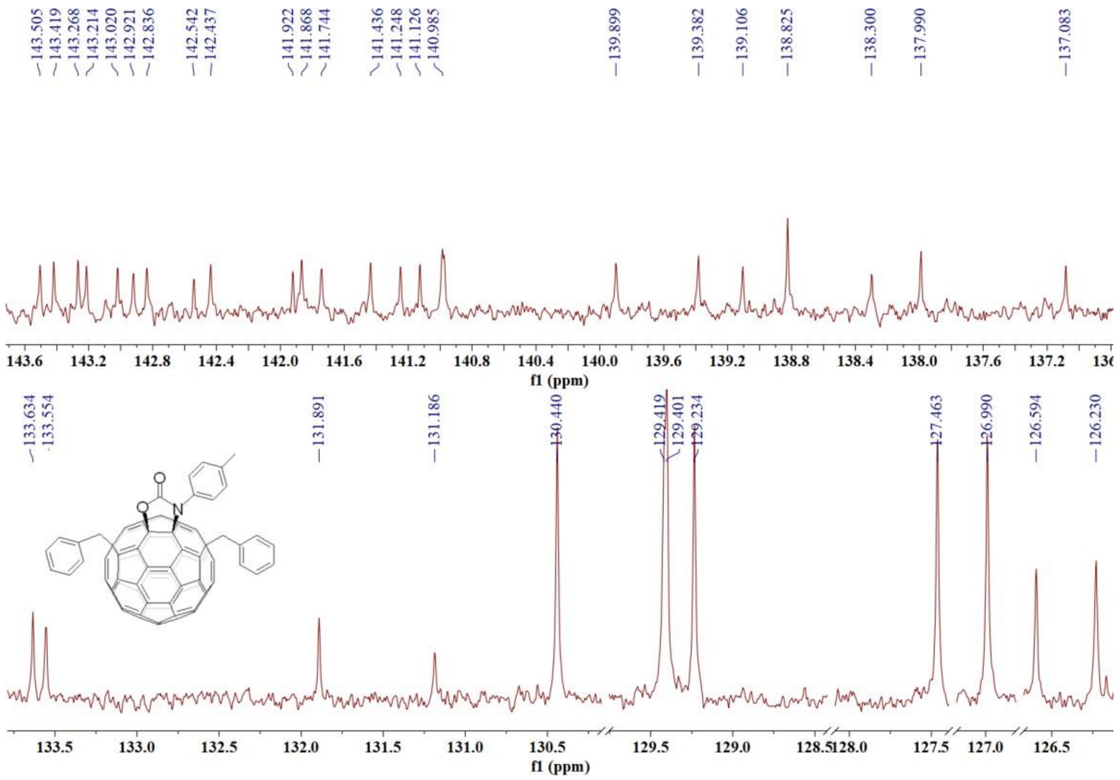

Figure S14. Expanded ^{13}C NMR (101 MHz, CS_2 with DMSO-d_6 as the external deuterium lock) ofcompound **4**.

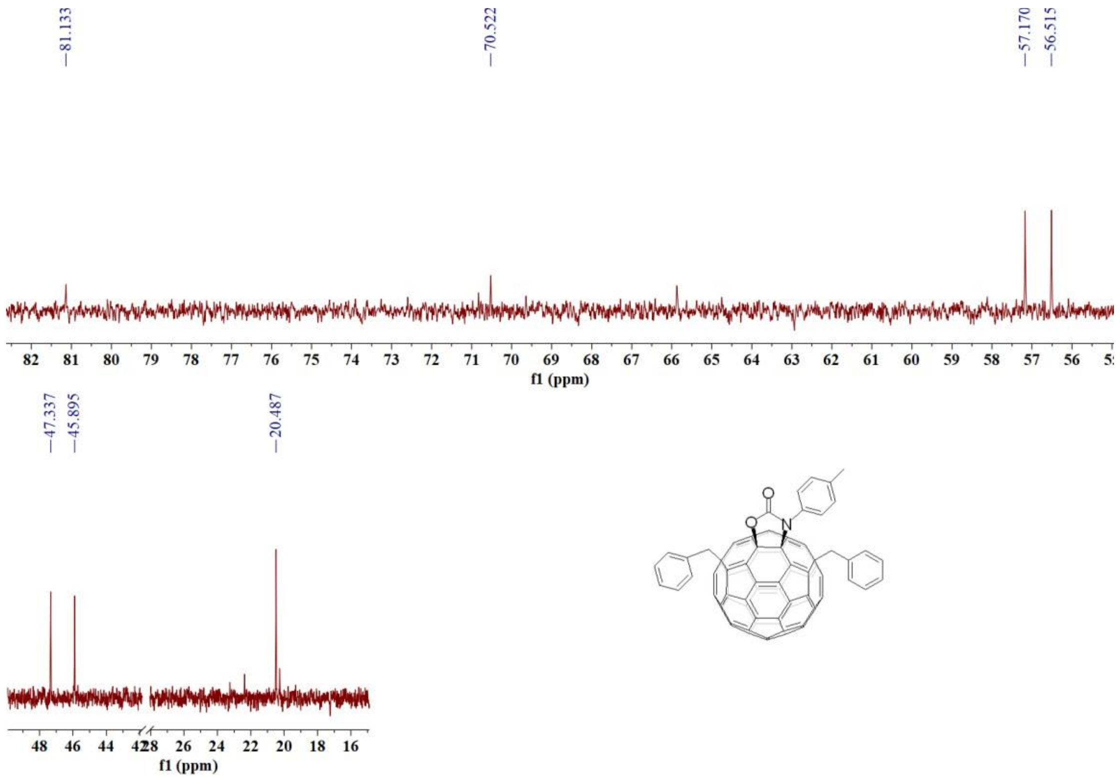

Figure S15. Expanded ^{13}C NMR (101 MHz, CS_2 with DMSO-d_6 as the external deuterium lock) ofcompound **4**.

4. UV-vis spectra of compounds 1–4 and PCBM

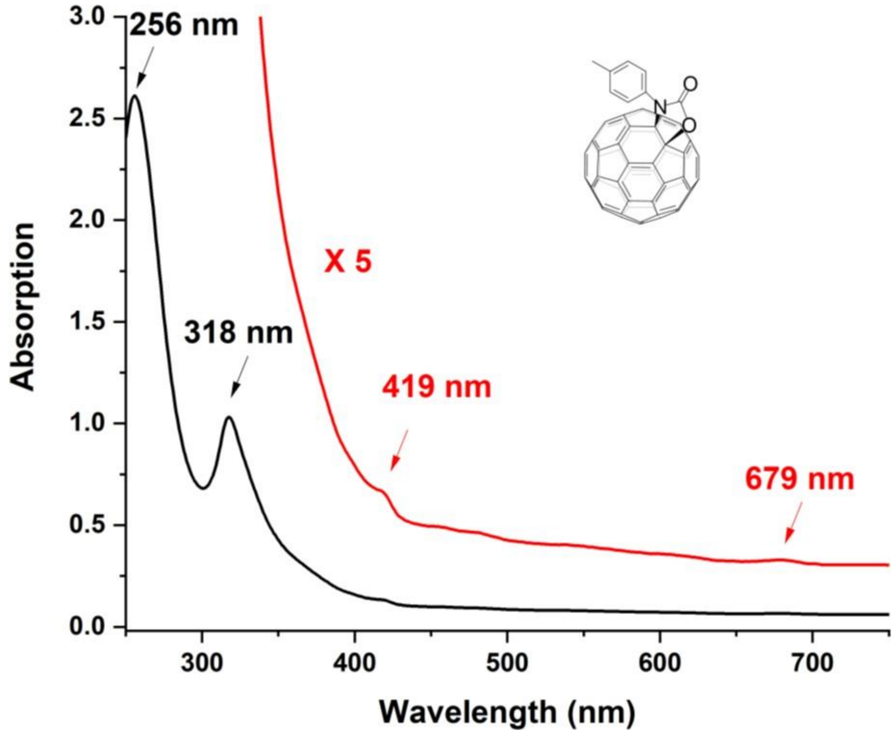

Figure S16. UV-vis spectrum of compound **1** in $CHCl_3$.

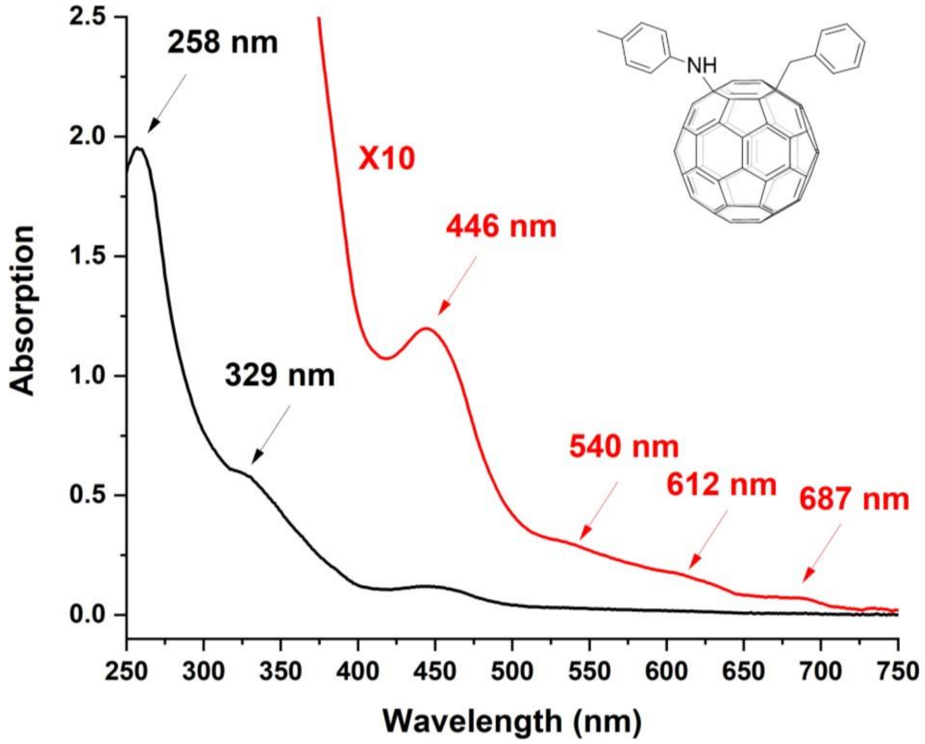

Figure S17. UV-vis spectrum of compound **2** in $CHCl_3$.

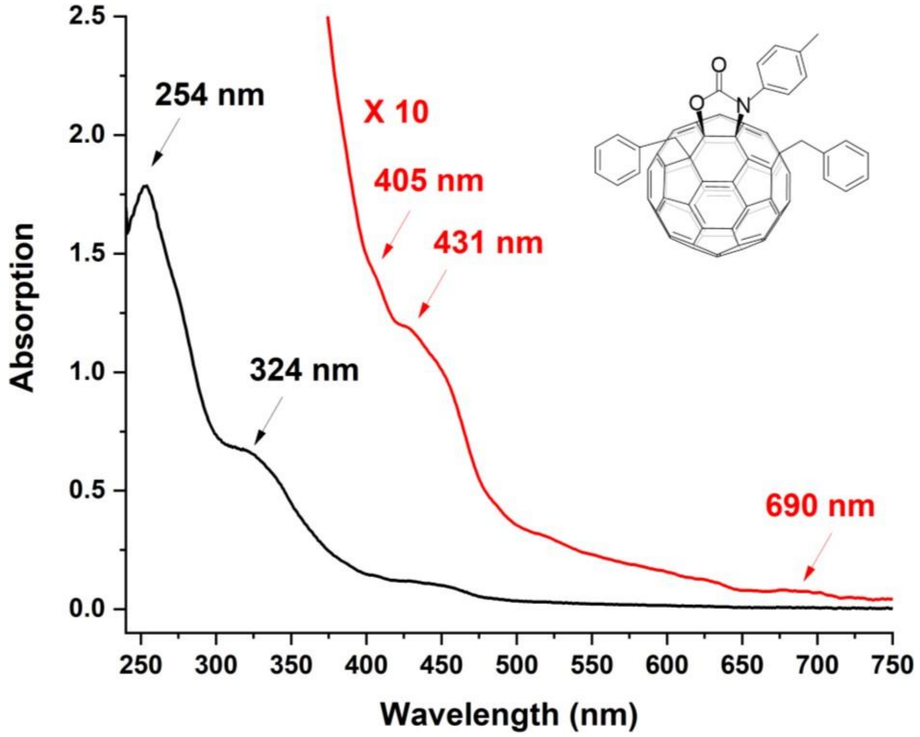

Figure S18. UV-vis spectrum of compound **3** in $CHCl_3$.

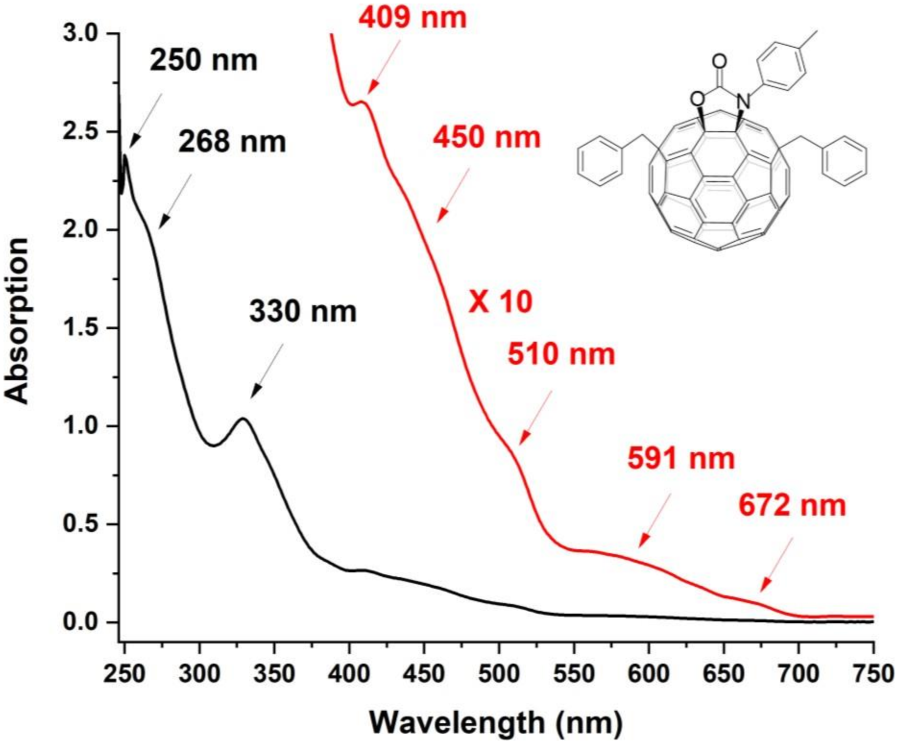

Figure S19. UV-vis spectrum of compound **4** in $CHCl_3$.

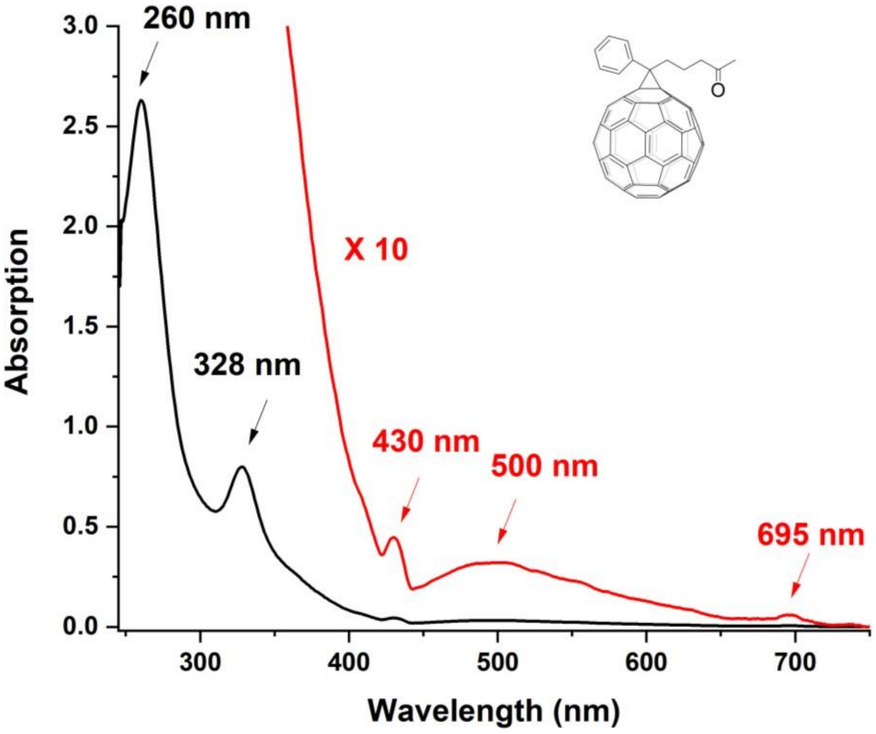

Figure S20. UV-vis spectrum of PCBM in $CHCl_3$.

5. CVs of dianion 1^{2-} and compounds 2–4

In Figure S21, the CV of dianion $\mathbf{1}^{2-}$ showed the different electrochemical properties compared to neutral compound **1**. In Figure S22, the first redox of compound **2** was quasi-reversible, and the second and third redoxes were reversible. In Figure S23, the first redox of compound **3** was quasi-reversible, while the other redoxes were all irreversible. In Figure S24, all redoxes of compound **4** were irreversible. In Figure S25, all redoxes of PCBM were reversible. Due to the different properties of these compounds, their wave heights

and reversibilities were not the same in CV. Therefore, the E_1 values in Table 1 in the main text were taken from their DPV measurements. The asterisks label the ferrocene/ferrocenium.

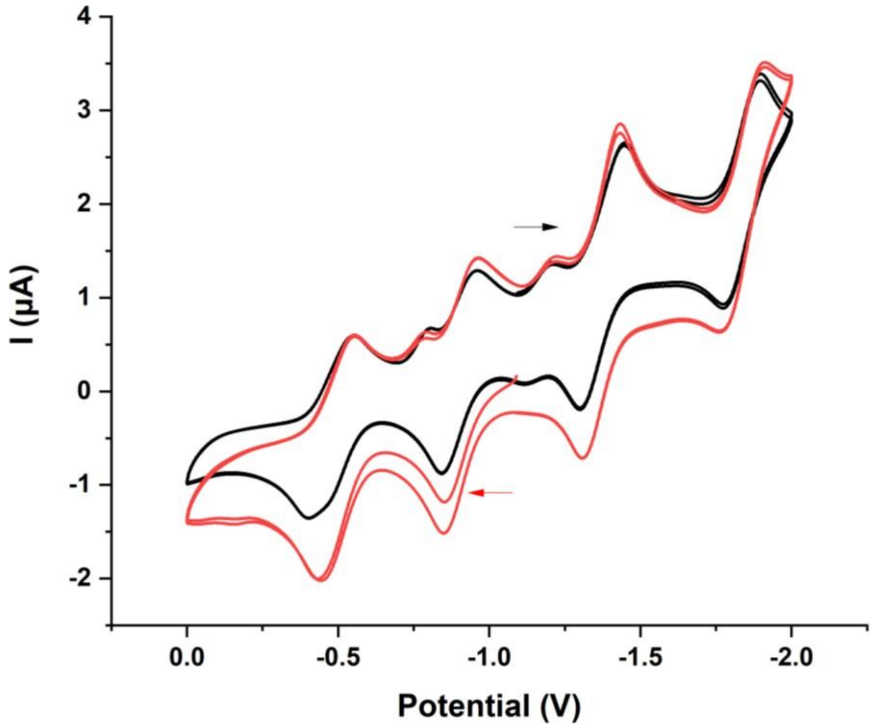

Figure S21. CV of dianion $\mathbf{1}^{2-}$ (1.0 mM) recorded in 1,2-$C_6H_4Cl_2$ containing 0.1 M TBAP at 15 °C. The parameters of CV: scan rate: 50 mV s^{-1}; initial potential: –1.09 V; initial scan polarity: negative or positive.

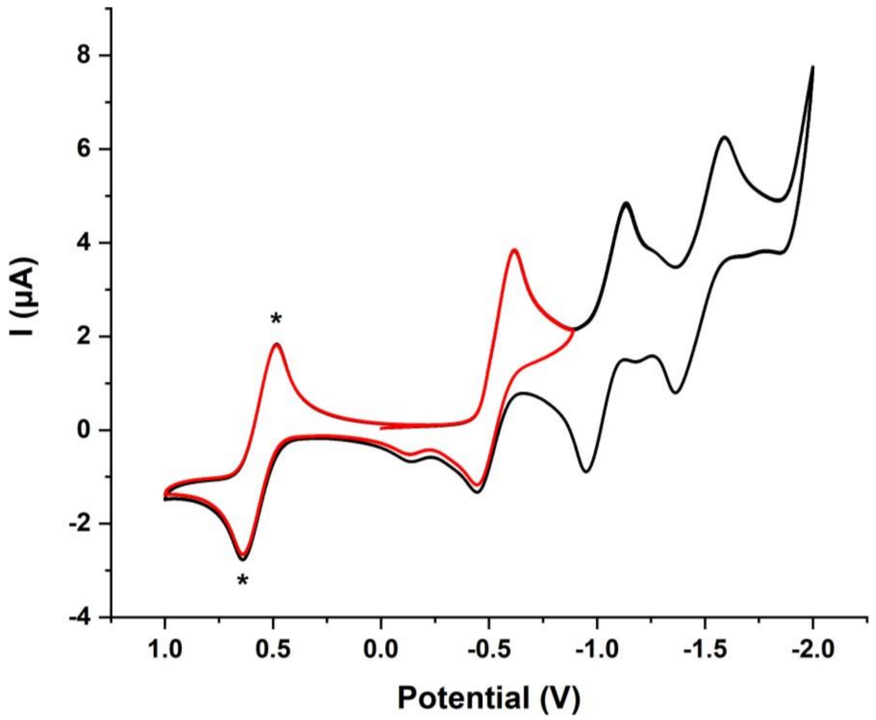

Figure S22. CV of compound **2** (1.0 mM) recorded in 1,2-$C_6H_4Cl_2$ containing 0.1 M TBAP with ferrocene(1.0 mM) as reference at 25 °C. The parameters of CV: scan rate: 50 mV s^{-1}; initial potential: 0.0 V; initial scan polarity: negative. The asterisks label the ferrocene/ferrocenium.

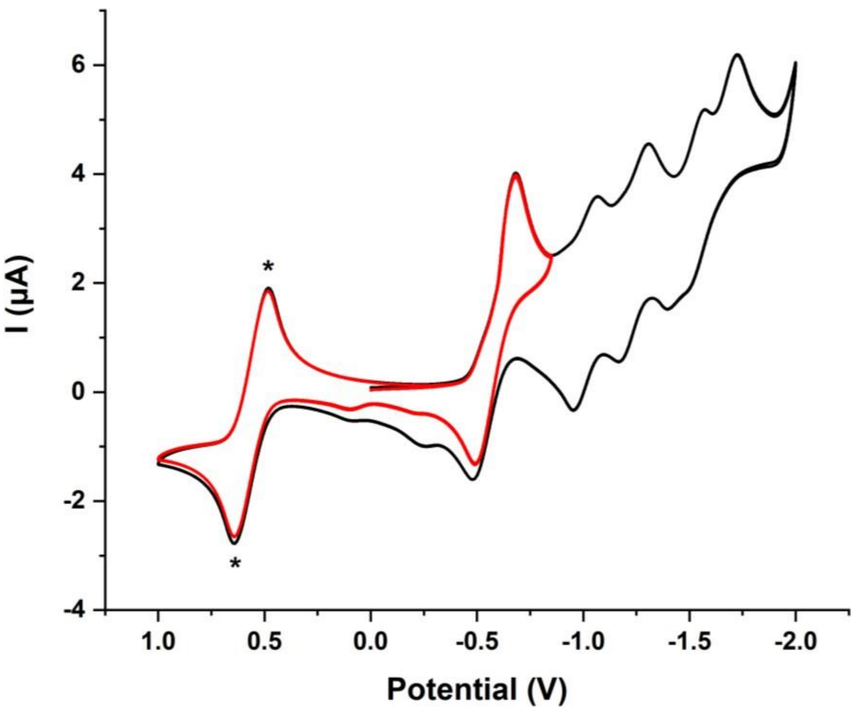

Figure S23. CV of compound **3** (1.0 mM) recorded in 1,2-$C_6H_4Cl_2$ containing 0.1 M TBAP with ferrocene(1.0 mM) as reference at 25 ^{o}C. The parameters of CV: scan rate: 50 mV s^{-1}; initial potential: 0.0 V; initial scan polarity: negative. The asterisks label the ferrocene/ferrocenium.

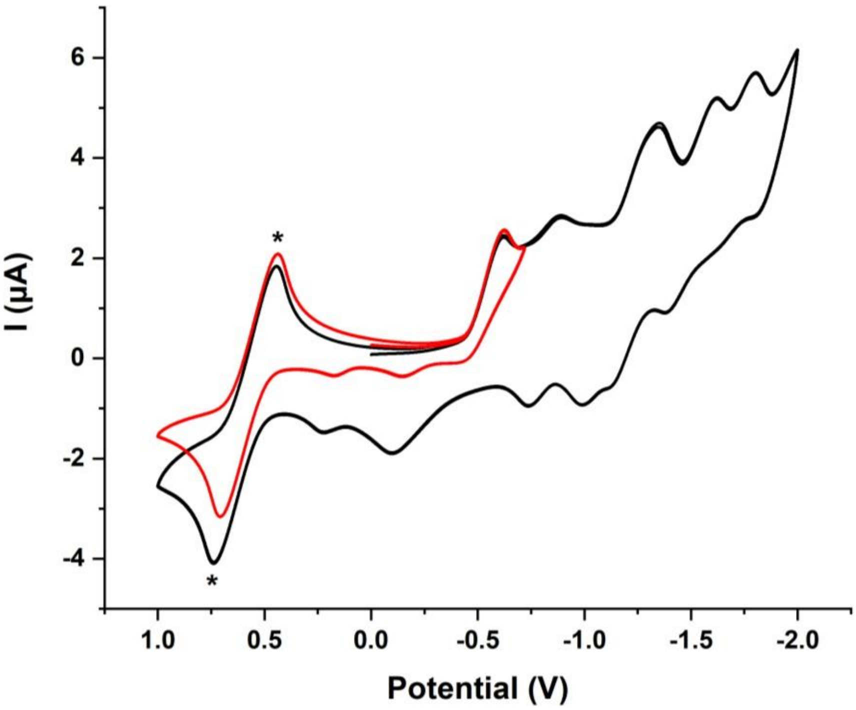

Figure S24. CV of compound **4** (1.0 mM) recorded in 1,2-$C_6H_4Cl_2$ containing 0.1 M TBAP with ferrocene(1.0 mM) as reference at 25 ^{o}C. The parameters of CV: scan rate: 50 mV s^{-1}; initial potential: 0.0 V; initial scan polarity: negative. The asterisks label the ferrocene/ferrocenium.

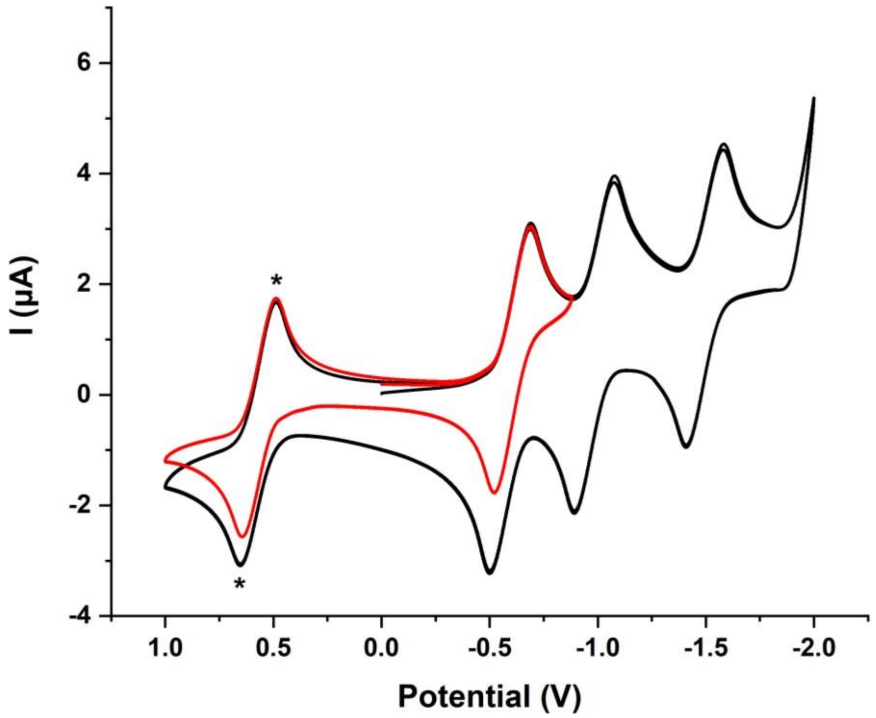

Figure S25. CV of PCBM (1.0 mM) recorded in 1,2-$C_6H_4Cl_2$ containing 0.1 M TBAP with ferrocene (1.0 mM) as reference at 25 °C. The parameters of CV: scan rate: 50 mV s^{-1}; initial potential: 0.0 V; initial scanpolarity: negative. The asterisks label the ferrocene/ferrocenium.

6. X-Ray single-crystal data of compound 3

Table S1. Crystal Data and Structure Refinement for Compound **3.**

Identification code	2163434
Empirical formula	C83H21NO2S2
Formula weight	1128.13
Temperature/K	293(2)
Crystal system	triclinic
Space group	P−1
a/Å	10.3287(7)
b/Å	14.6619(8)
c/Å	15.9784(7)
α/°	89.711(4)
β/°	87.151(4)
γ/°	88.867(5)
Volume/Å^3	2416.3(2)
Z	2
ϱcalcg/cm^3	1.551
μ/mm^{-1}	1.506
F(000)	1148.0
Crystal size/mm^3	0.24 × 0.23 × 0.21
Radiation	CuKα (λ = 1.54184)
2θrange for data collection/°	8.174 to 150.64
Index ranges	−9 ≤ h ≤ 12, −17 ≤ k ≤ 17, −19 ≤ l ≤ 19
Reflections collected	14524
Independent reflections	9094 [R_{int} = 0.0551, R_{sigma} = 0.0593]
Data/restraints/parameters	9094/0/794
Goodness-of-fit on F^2	1.059
Final R indexes [I>=2σ (I)]	R_1 = 0.0956, wR_2 = 0.2686
Final R indexes [all data]	R_1 = 0.1388, wR_2 = 0.3074
Largest diff. peak/hole / e Å^{-3}	0.33/-0.78
